# Hierarchical supramolecular co-assembly formation employing multi-component light-harvesting charge transfer interactions giving rise to long-wavelength emitting luminescent microspheres†

**DOI:** 10.1039/d2sc02097a

**Published:** 2022-05-31

**Authors:** Tumpa Gorai, June I. Lovitt, Deivasigamani Umadevi, Gavin McManus, Thorfinnur Gunnlaugsson

**Affiliations:** School of Chemistry and Trinity Biomedical Sciences Institute (TBSI), Trinity College Dublin, The University of Dublin Dublin 2 Ireland gorait@tcd.ie gunnlaut@tcd.ie; Advanced Materials and BioEngineering Research (AMBER) Centre, Trinity College Dublin, The University of Dublin Dublin 2 Ireland; Department of Chemistry, Indian Institute of Technology Palakkad (IITPKD) Palakkad-678557 Kerala India; School of Biochemistry and Immunology, Trinity Biomedical Sciences Institute (TBSI), Trinity College Dublin, The University of Dublin Dublin 2 Ireland; Synthesis and Solid State Pharmaceutical Centre (SSPC) Ireland

## Abstract

Charge transfer (CT) interaction induced formation of a hierarchical supramolecular assembly has attracted attention due to its wide diversity of structural and functional characteristics. In the present work, we report the generation of green luminescent microspheres from the charge transfer interaction induced co-assembly of a bis-naphthyl dipicolinic amide (DPA) derivative with tetracyanobenzene (TCNB) for the first time. The properties of these self-assemblies were studied both in solution and the solid-state using spectroscopic and a variety of microscopy techniques. The X-ray crystal structure analysis showed a mixed stack arrangement of DPA and TCNB. The molecular orbital and energy level calculations confirm the charge transfer complex formation between DPA and TCNB. Furthermore, energy transfer was observed from the green luminescent CT complex to a red-emitting dye, pyronin Y, in the microsphere matrix, leading to the formation of a light-harvesting tri-component self-assembly.

## Abstract 2

Green luminescent microspheres, generated *via* charge-transfer interaction-induced hierarchical co-assembly of a bis-naphthyl dipicolinic amide derivative and tetracyanobenzene, transfer energy to long-wave emitting dye.

## Graphical abstract

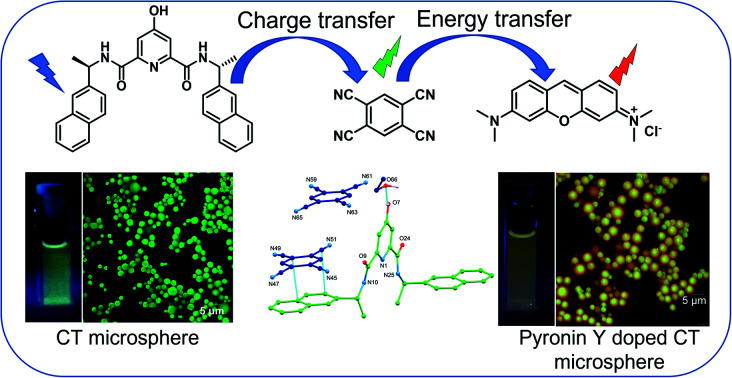

## Introduction

Supramolecular organization of multiple components by directional non-covalent interactions is a fascinating approach to develop well-defined hierarchical self-assembled superstructures with unique and targeted functional properties.^1^ The attractiveness of such systems is that they allow crucial spatial re-organization of self-assembled materials by structural and compositional variation without the necessity of complex synthesis. The co-assembled materials are promising candidates for exploring unique properties and their applications such as in tunable luminescence,^1^ light-harvesting,^2^ chiroptics,^3^ optoelectronics,^4^ catalysis,^5^*etc.* We have particular interest in the formation and characterisation of functional luminescent materials.^6^ This is a fast-growing area of research, and there have been numerous recent reports on the development of such systems, using, for instance, the Förster resonance energy transfer (FRET) mechanism in the generation of supramolecular light-harvesting self-assemblies,^7^ in the formation of emissive hydrogels and organogels,^8^ luminescent MOFs,^9^ and higher order nano-assemblies,^10^ to name just a few.

Supramolecular light-harvesting assemblies are of particular interest to our research, the mechanism of which often mimics natural photosynthetic systems.^11^ Charge transfer (CT) complex-based co-assemblies and co-crystals are gaining attention for their application in bioimaging, organic optoelectronic materials, sensors, stimuli-responsive materials, *etc.*^12,13,15,16^ CT complexes consist of electron-rich donor and electron-deficient acceptor ligands and are organized by favourable non-covalent supramolecular interactions, preferably π–π stacking.^13,14^ They have been used in the formation of fluorescent microdumbbells,^15*a*^ microcapsules,^15*b*^ organogels,^15*c*^ and microtubes,^16*a*^ from CT interaction induced self-assemblies of various aromatic chromophores with tetracyanobenzene (TCNB) as the acceptor. However, there are no reports yet on the formation of fluorescent microspheres formed from a co-assembly of an organic chromophore with TCNB. While there are a few examples on the use of the energy transfer mechanism from one charge transfer (CT) complex to another CT complex, which were employed in the formation of such multiple component co-assembly structures,^16^ to the best of our knowledge, no reports have demonstrated the energy transfer from a CT complex to a fluorescent dye resulting in such material properties.

**Fig. 1:**
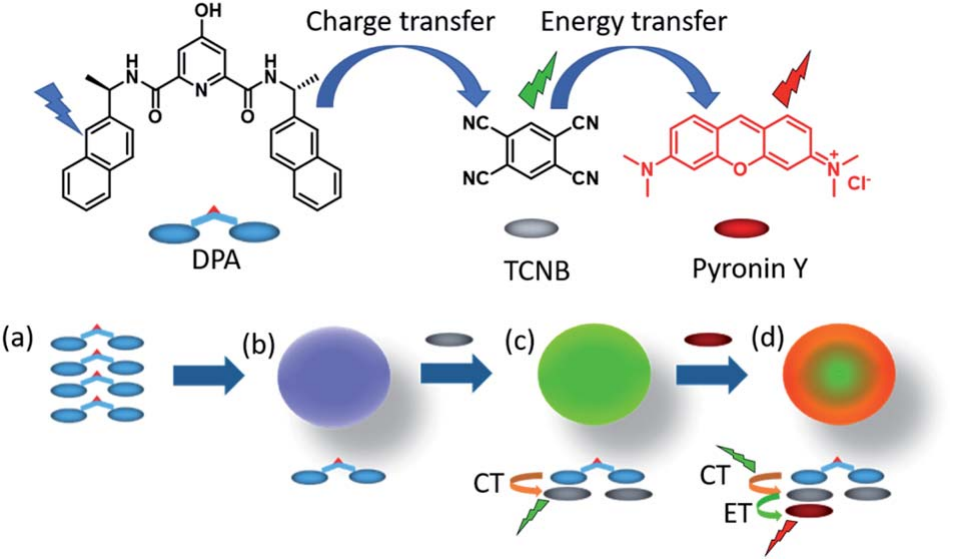
The chemical structures of DPA, TCNB and pyronin Y, and proposed schematic for (a and b) microsphere formation from the self-assembly of DPA, (c) green fluorescent microsphere formation by CT interaction induced co-assembly of DPA with TCNB in ACN/EtOH/water solvent, and (d) energy transfer (ET) from the green charge transfer (CT) complex to pyronin Y in the tri-component assembly in an ACN/EtOH/water solvent mixture.

In the work presented herein, we report the formation of luminescent microspheres from the co-assembly of a 4-hydroxy-*N*^2^,*N*^6^-bis((*R*)-1-(naphthalen-2-yl)ethyl)pyridine-2,6-dicarboxamide (DPA) derivative and TCNB. Furthermore, X-ray structural analysis showed that these green-emitting microspheres are the result of a CT interaction between the naphthalene unit of the DPA and TCNB. Moreover, we illustrated that the association of this green emissive assembly with the red fluorescent pyronin Y dye results in a ‘stepping stone’ energy transfer process that results in the formation of a red-emitting hierarchical supramolecular co-assembly possessing CT character (Fig. 1).

## Results and discussion

### Self-assembly studies of DPA in mixtures of organic–aqueous solutions

**Fig. 2:**
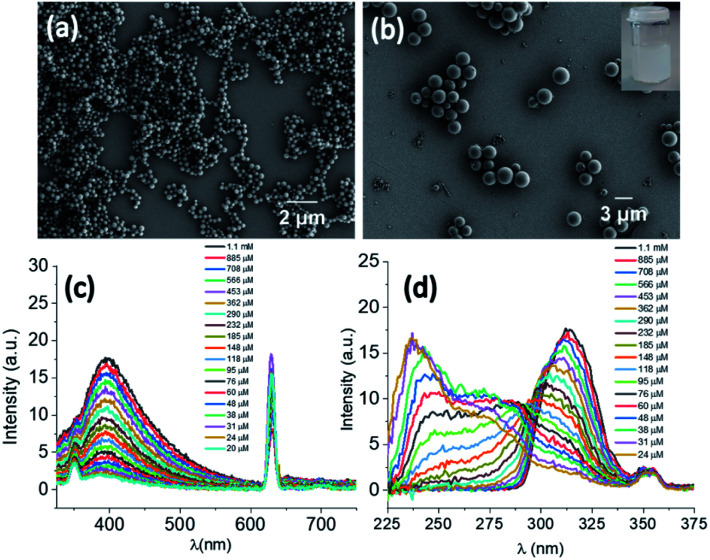
SEM images of the DPA derivative (a) immediately after preparation and (b) after 24 h of aging. (c) Emission spectra recorded for DPA (1.1 mM) solution on serial dilution in ACN/EtOH/H_2_O (10/40/50%) solvent for *λ*_ex_ of 315 nm. The peak at 630 nm is the overtone band. (d) Excitation spectra recorded for DPA (1.1 mM) solution for *λ*_em_ of 397 nm on serial dilution in ACN/EtOH/H_2_O (10/40/50%) solvent.

DPA was synthesized by a peptide coupling reaction between (*R*)-1-(2-naphthyl)-1-ethyl amine and chelidamic acid following earlier reports for similar compounds^17^ and characterized by standard characterization techniques *e.g.*, ^1^H and ^13^C NMR, HRMS, IR spectroscopy and elemental analysis (Section 4, ESI†). DPA had good solubility in solvents such as acetonitrile (ACN) or ethanol (EtOH) or a mixture of both solvents at room temperature. Addition of 60% water (3 mL) to the DPA (final conc. 1 mM) solution in an ACN/EtOH (20/80%)‡‡Since TCNB is not readily soluble in EtOH, 20% ACN was added to solubilize it. Therefore, both DPA and TCNB solutions were made in an ACN/EtOH (20/80%) mixture. mixture (2 mL) generates a suspension (Section 5, Fig. S4a, ESI†). Scanning electron microscopy (SEM) investigations of the material (upon dropcasting onto a silicon wafer) showed the formation of nanospheres with an average diameter of 282 ± 44 nm (Fig. 2a and S5, ESI†). Upon heating the solution to 50 °C, a clear solution was obtained. However, on cooling the suspension was re-formed. Further ageing of this solution at room temperature for 24 h led to a conversion to microspheres with a size range of 2.35 ± 0.38 μm (Fig. 2b and S4b and c and S6, ESI†).

The microsphere formation was investigated further under different solvent conditions, *e.g.*, the clear solution in ACN/EtOH (20/80%) (Fig. S7, ESI†) and ACN/EtOH/water (12/48/40%) (Fig. S8, ESI†) solvents. For both samples, the sphere formation was observed, although the size was found to be ≤1 μm in diameter. To understand the aggregation behaviour of DPA, absorption, emission and excitation spectra were also recorded on serial dilution of DPA (1100–20 μM) in an ACN/EtOH/water (10/40/50%) mixture. The absorption at 315 nm (Fig. S9c, ESI†) and emission spectral intensity for 315 nm excitation increased with increasing DPA concentration (Fig. 2c and S10, ESI†). On increasing DPA concentration, the excitation spectra showed a redshift with an intensity reduction of the excitation band at *λ*_max_ of 236 nm and *λ*_max_ of 275 nm at 20 μM concentration and a concomitant intensity rise of the band at *λ*_max_ of 315 nm recorded up to 1.1 mM concentration (Fig. 2d). The shift in the excitation band is due to the aggregation of DPA. Initially, the changes in the absorption at 275 nm (Fig. S9b, ESI†) followed the Beer–Lambert law with a concomitant enhancement in the fluorescence emission (Fig. S11, ESI†). However, above 60 μM concentration with increasing absorption, emission showed a steady decrease due to the bathochromic shift of the excitation spectrum.

### Self-assembly studies of DPA in the presence of TCNB in solution and the solid-state

**Fig. 3:**
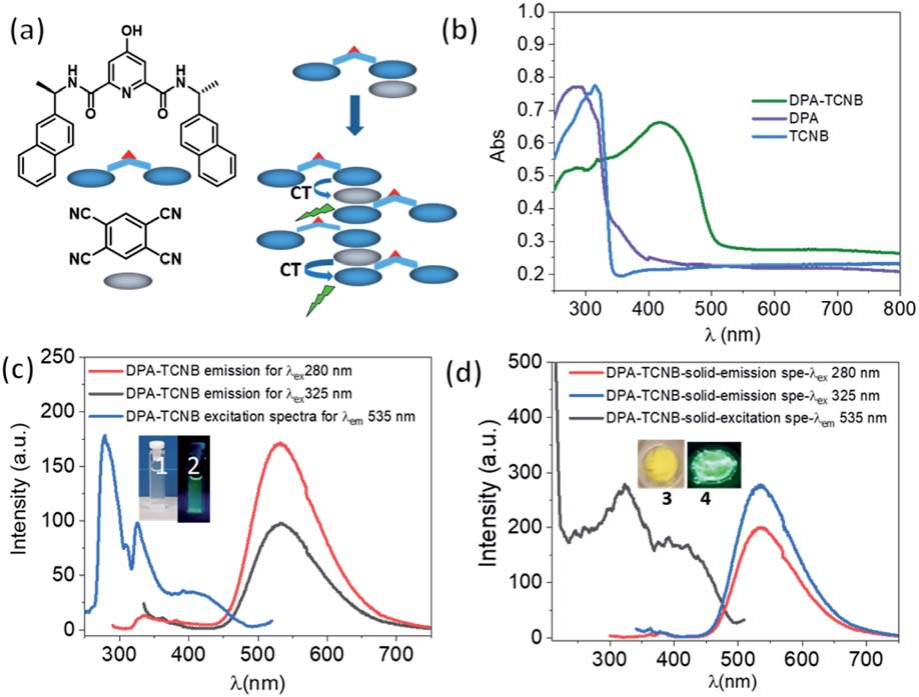
(a) Schematic for charge transfer complex formation from the DPA and TCNB co-assembly; (b) solid-state absorption spectra of only DPA, TCNB and DPA–TCNB CT co-assembled materials, respectively; (c) emission spectra for *λ*_ex_ of 280 nm and 325 nm and excitation spectrum for *λ*_em_ of 535 nm for the DPA–TCNB suspension (∼162 μM); inset pictures 1 and 2: DPA–TCNB CT suspension in daylight and under a 365 nm UV lamp, respectively; (d) solid-state emission spectra of DPA–TCNB for *λ*_ex_ of 280 nm and 325 nm, respectively, and excitation spectrum for *λ*_em_ of 535 nm; inset pictures 3 and 4: DPA–TCNB CT powder in daylight and under 365 nm UV light, respectively.

Having investigated the formation of the DPA microspheres in solution, we next explored the formation of a co-assembly between DPA and TCNB. As outlined above, CT interaction induced formation of co-crystals is an interesting area of research. In this context, we investigated the CT complex formation between DPA and TCNB spectroscopically. Indeed, on addition of TCNB (5 mM) to a solution of DPA (5 mM) in the ACN/EtOH (20/80%) mixture, a light-yellow solution formed. Upon increasing the percentage of water, the colour intensified, and a light-yellow suspension was formed in ACN/EtOH/water (10/40/50%) solvent (Fig. S12, ESI†). The UV-Vis absorption spectra of DPA–TCNB (5.8 mM each) (Fig. S13, ESI†) solution in the ACN/EtOH/water (12/48/40%) mixture showed the appearance of a low-intensity new band within the 330–500 nm window upon the formation of a yellow solution, indicating the association between two components and the formation of a CT band. When the DPA concentration was reduced, a higher percentage of water was required to generate the suspension. The absorption, fluorescence emission and excitation spectra (Fig. S14, ESI†) were also recorded concomitantly for the DPA–TCNB (1 mM each) co-assembly with increasing water percentage. No changes were observed in the emission wavelength until the formation of the yellow suspension, when a shift to longer wavelengths was observed (Fig. S14b, ESI†). In solution, this emission band was not observed upon recording the emission arising from the DPA suspension or the TCNB solution (Fig. S15, ESI†) alone. We assigned the generation of the green emission (which was clearly visible to the naked eye) to a CT emission that is caused by aggregation of the two components.^16*a*^ A control experiment for the emission of DPA alone with increasing water percentage showed a slight variation in intensity (Fig. S16, ESI†); however, no spectral shift was observed. When the solid-state absorption was recorded for DPA, TCNB and the DPA–TCNB co-assembly (Fig. 3b), in contrast to what was seen for DPA or TCNB alone, and as was observed in solution (Fig. S13, ESI†), an additional spectral band was seen for the DPA–TCNB co-assembly, within the 350–500 nm range. Furthermore, under UV-light, the solid gave rise to green fluorescence, which was visible to the naked eye.

The emission and excitation spectra recorded for both the suspension and the isolated solid DPA–TCNB samples showed bands that were structurally similar, with the fluorescence emission occurring within the 450–700 nm range with *λ*_max_ at 535 nm and excitation spectra in the 270–500 nm range (Fig. 3c and d). The excitation spectrum for the diluted suspension (162 μM) has *λ*_max_ at 280 nm and 325 nm, and the concentrated suspension (1 mM) has *λ*_max_ at 330 nm with a low-intensity band at 280 nm (Fig. S17, ESI†). For the solid DPA–TCNB sample, a broad excitation spectrum was observed with *λ*_max_ of 325 nm (Fig. 3d), which indicates stronger aggregation in the concentrated suspension and solid-state compared to the diluted suspension. The fluorescence excitation bands in the 270–500 nm range for both suspension and solid samples matched well with the absorption spectra of the DPA–TCNB solid within the same region (Fig. S18, ESI†), indicating that the emission was unambiguously due to the CT interaction of DPA with TCNB.

When the emission spectra were recorded for DPA–TCNB with fixed DPA (1 mM) and increasing TCNB (1–4 mM) concentration, no significant increase in the emission intensity was observed (Fig. S19–S21, ESI†). Therefore, for the remaining experiments, DPA and TCNB were used in a 1 : 1 ratio.

**Fig. 4:**
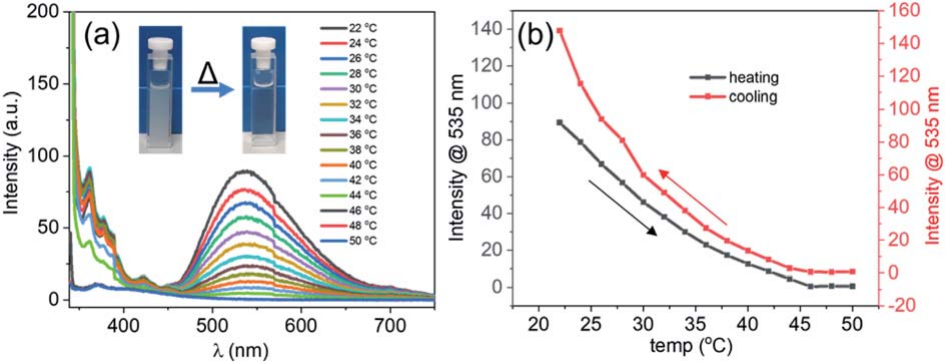
(a) Emission spectra recorded for the DPA–TCNB (1 mM each) CT suspension in ACN/EtOH/water (8 : 32 : 60%) on gradual heating from 22 °C to 50 °C; (b) emission changes at 535 nm for the DPA–TCNB CT suspension with increasing and decreasing temperature.

As was mentioned above, the formation of the DPA spheres was found to be reversibly temperature-dependent. To understand if such a thermo-responsive behaviour was also observed for the DPA–TCNB samples, a heating–cooling experiment was performed on a freshly prepared suspension (1 mM), where the emission spectra were recorded as a function of temperature. The overall results are shown in Fig. 4. Here, the CT induced yellow suspension (DPA and TCNB (1 mM each) in ACN/EtOH/water (8/32/60%)) turned into a clear solution upon heating from 20 to 50 °C. The concomitant changes in the emission are shown in Fig. 4a, while Fig. 4b shows that at 46 °C the emission at 535 nm was completely quenched. Gratifyingly, these changes were fully reversible as upon cooling (Fig. S22, ESI† and Fig. 4b), the emission spectra showed that the CT band reappeared below *ca.* 46 °C. These observations signify the reversible thermo-responsive behaviour of the suspension.

### Morphological and confocal fluorescence microscopy examinations of the DPA–TCNB co-assembly

**Fig. 5:**
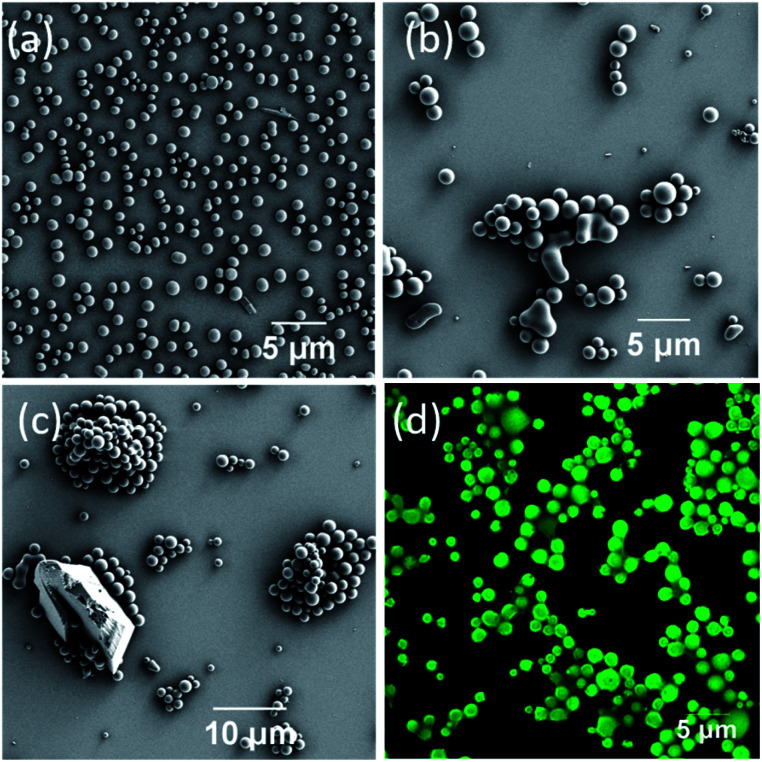
SEM images of the dropcast and dried DPA–TCNB CT complex: (a) clear solution in ACN/EtOH (20/80%) and (b and c) DPA–TCNB suspension in ACN/EtOH/water (8/32/60%); (d) confocal microscopy image of the dropcast and dried DPA–TCNB CT suspension recorded under 405 nm laser excitation.

Having illustrated the emission properties of the DPA–TCNB assembly in solution, we next probed the morphological properties of this system. Here, SEM investigation of DPA–TCNB solution in ACN/EtOH (20/80%) showed, as was the case of the DPA (Fig. S7, ESI†), a well-separated spherical morphology (Fig. 5a and S23, ESI†) for samples that were dropcast and dried. The SEM images of the DPA–TCNB suspension in ACN/EtOH/water (8/32/60%) showed aggregated spheres with crystalline particles in some regions (Fig. 5b and c and S24, ESI†), which may be due to either unreacted TCNB crystals or the initiation of CT crystal growth.

As we had determined above, the CT co-assembly was emissive in suspension, giving rise to green emission. Using confocal fluorescence microscopy, we demonstrated, in Fig. 5d, that the spherical co-assembly morphology also gave rise to green emission under 405 nm laser excitation. Furthermore, we demonstrated that in solution the particle size increased over time, and the time-dependent fluorescence and SEM measurement corroborated this (Fig. S25, ESI†). The encapsulation of TCNB in the interior of the microsphere matrix minimized the solvent-induced quenching effect, as well as the formation of an extended CT network in the larger diameter microsphere matrix, which led to an increase in emission with increasing particle size. Therefore, as the particles grow from nanospheres to microspheres after 24 hours, the emission showed a sharper rise, followed by slower rises on the second and third days as particle growth slowly reached equilibrium (Fig. S25a†).

### Solid-state crystallographic and powder diffraction examinations of the DPA–TCNB co-assembly

Having demonstrated that the DPA–TCNB gave rise to green emission, we investigated the solid-state behaviour of the assembly to probe the supramolecular interactions causing the CT excited state properties. Gratifyingly, single crystals of the DPA–TCNB charge transfer complex suitable for X-ray diffraction analysis were grown from dissolution in an ACN/EtOH/H_2_O (12/48/40%) mixture after standing at room temperature for two weeks.

**Fig. 6:**
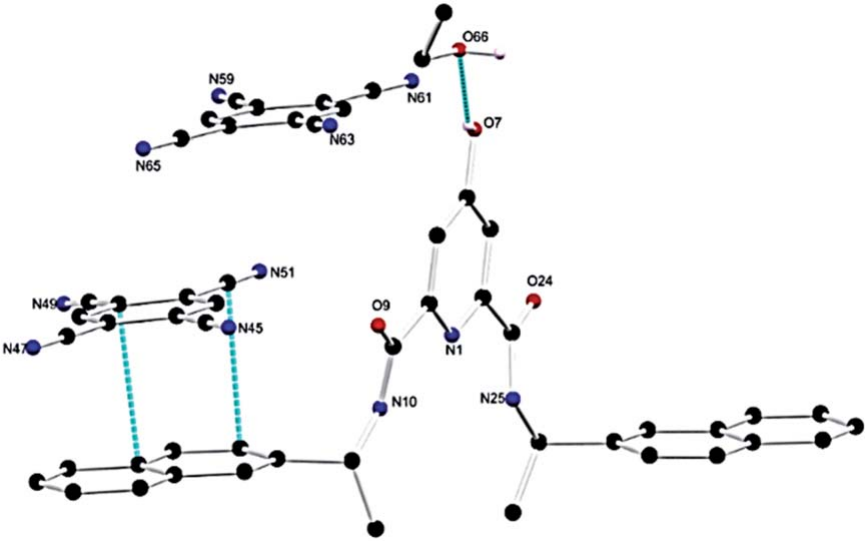
Structure of the DPA–TCNB CT complex with heteroatom labelling scheme. Selected hydrogen atoms are omitted for clarity.

**Fig. 7:**
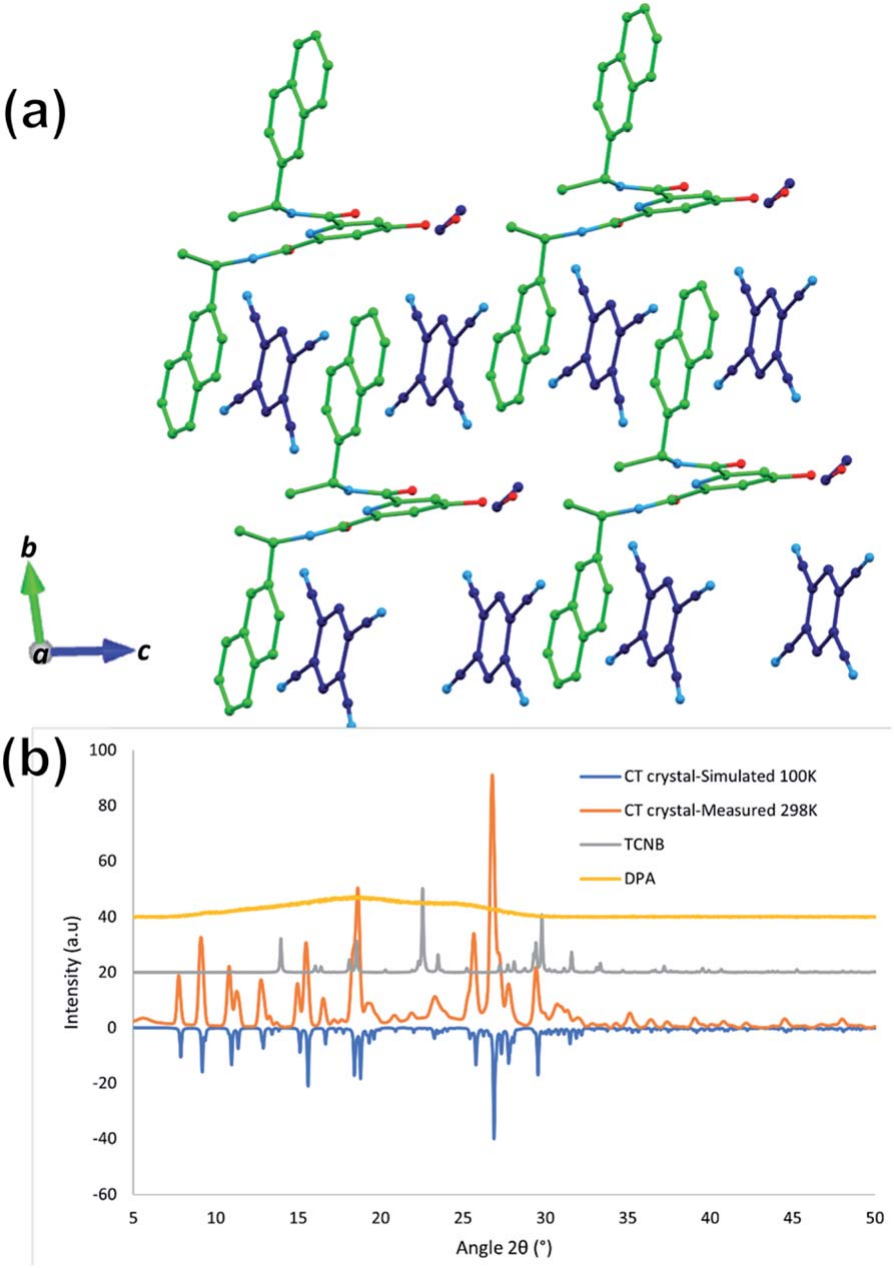
(a) Extended structure of the CT complex featuring numerous π⋯π stacking interactions between the naphthalene ring and TCNB guest molecules. Hydrogen atoms are omitted for clarity. DPA's C atoms are coloured in green and TCNB's C atoms are coloured in deep blue; O is in red, and N is in sky blue for both DPA and TCNB. (b) X-ray powder diffraction pattern for the DPA–TCNB CT complex collected at 298 K compared to those of DPA and TCNB and the calculated pattern from the single crystal dataset of the DPA–TCNB co-crystal at 100 K.

The X-ray diffraction analysis of the yellow block crystals was obtained and provided a structural model in the chiral triclinic space group *P*1 (crystallographic data and refinement parameters are summarised in Table S1, ESI†). The asymmetric unit contained one complete DPA molecule with one fully occupied ethanol molecule and two fully occupied TCNB molecules per DPA molecule as illustrated in Fig. 6. The extended structure of the DPA–TCNB crystal is supported through both hydrogen bonds and π⋯π interactions, as illustrated in Fig. 7a, which shows that the TCNB guest molecule has π⋯π stacking interactions with two adjacent DPA molecules through the naphthalene moieties. TCNB molecules are sandwiched in stacking π⋯π interactions with the distance between rings (C16 to C21 and C38 to C43) is 3.364(3) Å (see also Fig. S27, ESI†). There are also weaker C–H⋯O interactions between adjacent DPA moieties with O9–H26–C26 distance of 3.261(4) Å and angle of 144.9(2)°. There is a hydrogen bond between naphthalene O7–H7 and guest ethanol molecule oxygen O66, with an O7 to O66 distance of 2.665 Å at an angle of 134(4)°. To further visualise intermolecular interactions in the extended structure, Hirshfeld surfaces and resultant fingerprint plots were calculated illustrating the strong π⋯π interactions and C–H⋯O interactions between moieties in this structure (Fig. S28, ESI†). Hence the alternate stacking between donor DPA and acceptor TCNB and hydrogen bonding with ethanol provides the system with the directional organization for crystallization.

The powder X-ray diffraction (PXRD) pattern showed that DPA itself is amorphous in nature, and the PXRD pattern of DPA–TCNB indicates the presence of a single crystalline phase, which matches well with the simulated pattern obtained from the single crystal dataset at 100 K (Fig. 7b). TCNB has one distinct phase with sharp peaks in the diffraction pattern compared to that of the CT crystal.

### Additional analysis of the DPA–TCNB co-assembly

**Fig. 8:**
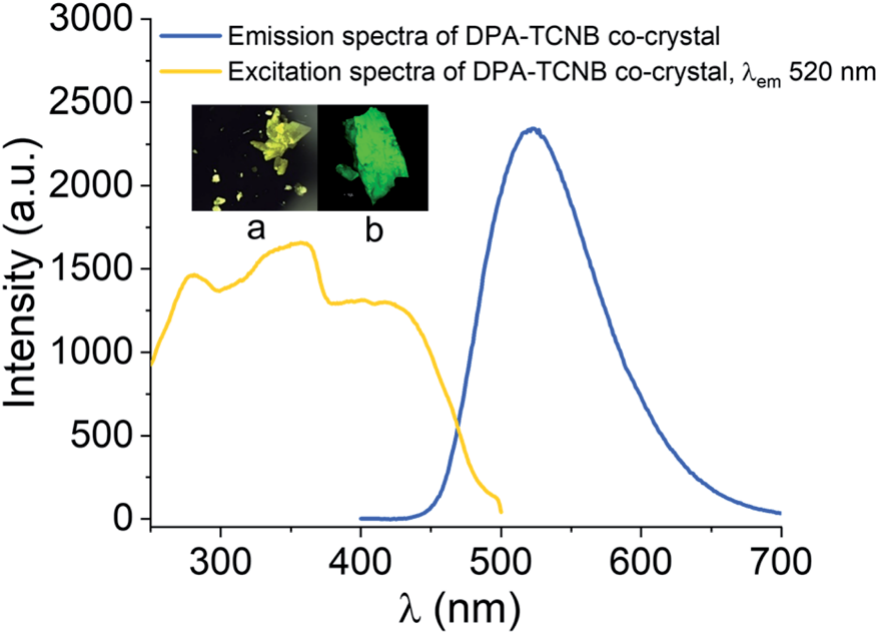
Emission spectrum for *λ*_ex_ of 350 nm and excitation spectrum for *λ*_em_ of 520 nm for the DPA–TCNB co-crystal; inset pictures: (a) optical and (b) confocal microscopy images of the DPA–TCNB co-crystal.

We have also recorded confocal images for the yellow co-crystals analysed here, which showed green emission (Fig. 8, inset pictures (a) and (b)). The emission maximum was slightly blue shifted with *λ*_max_ at 520 nm compared to the microsphere powder sample with *λ*_max_ at 535 nm, and excitation spectra recorded for crystals showed a similar CT band to sphere samples (Fig. S18†), proving that the emission from the sphere is solely due to charge-transfer interaction between the DPA ligand and TCNB.

Thermogravimetric analysis (TGA) was carried out for DPA–TCNB powder and DPA–TCNB crystal samples. The TGA curves showed a slight reduction in mass at around 100 °C due to the evaporation of solvent molecules. The changes in the TGA curves for the co-assembled powder and co-assembled crystal were well consistent with the mixed pattern in the TGA curves observed for the DPA ligand and TCNB alone (Fig. S29 and S30, ESI†).

We also recorded the NMR spectra for DPA–TCNB powder materials imaged in the SEM experiments. These showed the coexistence of ligand and TCNB (Fig. S31, ESI†). The IR spectra for the DPA–TCNB sample showed peaks at 3114 cm^−1^, 3084 cm^−1^, and 2247 cm^−1^ which correspond to the TCNB peaks, and peaks at 3284 cm^−1^, 2978 cm^−1^, and 2933 cm^−1^ which are consistent with DPA peaks in similar regions (Fig. S32, ESI†).

### Molecular orbital and energy level calculations

**Fig. 9:**
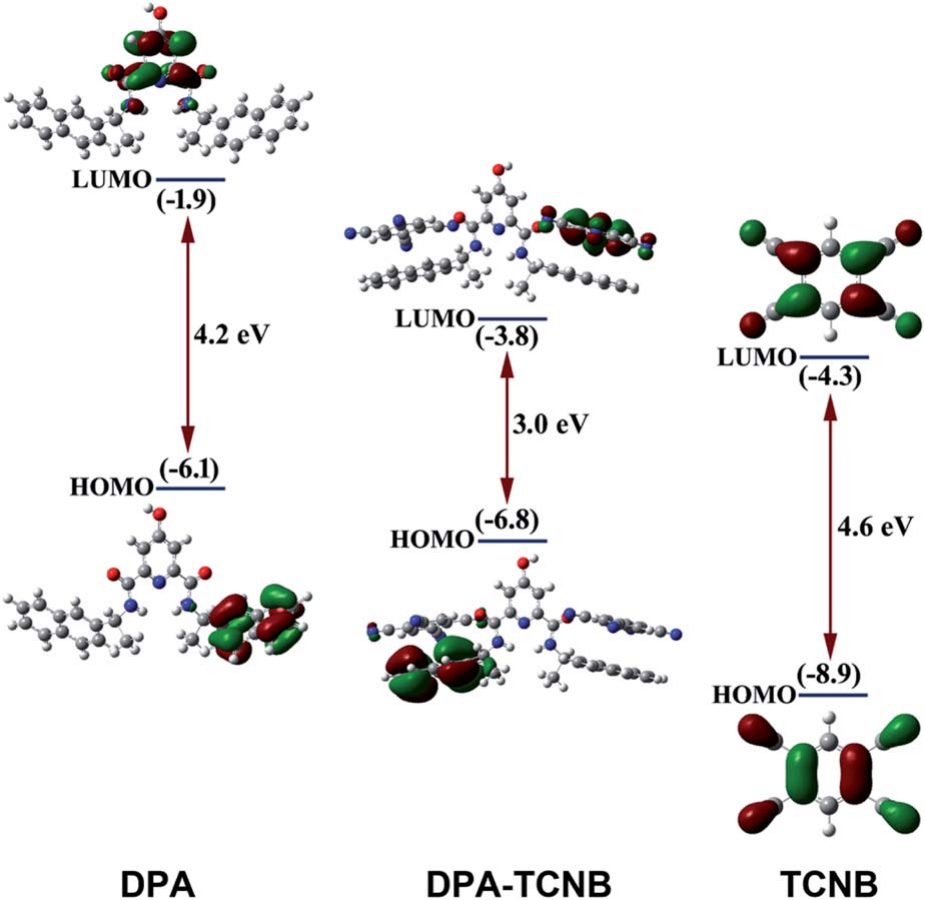
Overview of the calculated energy level diagram and MO diagram of DPA, TCNB and the DPA–TCNB complex obtained by employing density functional theory. Orbital plots were generated with a contour value of 0.04.

Molecular orbital (MO) and energy levels were calculated for the DPA, the TCNB and the DPA–TCNB charge transfer assembly, by employing density functional theory (DFT). Fig. 9 depicts the optimised geometry and the frontier orbital energies of DPA, TCNB and their complex. It is evident from the figure that the lowest unoccupied molecular orbital (LUMO) of the complex (−3.8 eV) lies closer to the acceptor LUMO (−4.3 eV), while the highest occupied molecular orbital (HOMO) (−6.8 eV) of the complex is closer to the donor HOMO (−6.1 eV). From the MO diagram, it is evident that the HOMO of the DPA + TCNB complex is mainly located on the DPA–naphthyl moiety and the LUMO is more localised in the TCNB moiety which confirmed the charge-transfer complex formation between DPA and TCNB *via* π–π stacking type of interactions.

### Formation of a long-wavelength emitting PYY–DPA–TCNB tri-component system

Having determined the CT nature of the DPA–TCNB co-assembly, and carried out the above calculations, we further explored the energy transfer properties of the assembly by co-doping this assembly with an organic molecule that could absorb the energy from the CT excited state, and emit at a long wavelength, as depicted in Fig. 1. This would result in a three-component co-assembly, functioning through initial CT, followed by sensitisation of the third component. With this in mind, we decided to use pyronin Y (PYY) and doped it into DPA–TCNB microspheres as described in Section 5, ESI.† One of the applications of pyronin Y is fluorescent staining of nucleic acids, especially RNA.^18^ Analysis of the emission spectrum of the DPA–TCNB charge transfer assembly showed good spectral overlap with the absorption spectrum of pyronin Y (Fig. S33, ESI†), this being a condition for pyronin Y to function as an energy transfer acceptor upon excitation of the DPA–TCNB assembly. Hence, based on this knowledge, we doped the DPA–TCNB spheres with pyronin Y at micromolar concentration (<2 mol%).

Emission spectra were recorded for pyronin Y in the DPA–TCNB–PYY tri-component assembly and compared with those of two alternative self-assemblies, as controls, namely pyronin Y encapsulated DPA spheres, and secondly, pyronin Y mixed with TCNB solution, as well as a solution containing only pyronin Y in an ACN/EtOH/water (8/32/60) mixture. High-intensity emission was observed for pyronin Y in the DPA–TCNB–PYY tri-component assembly. Furthermore, lower intensity pyronin Y centred emission was observed for the DPA–PYY sample, while negligible emission was observed for the TCNB–PYY sample and pyronin Y solution alone. This indicates that the emission intensity rise for pyronin Y in the tri-component assembly is due to an energy transfer from the green-emitting DPA–TCNB complex to pyronin Y (Fig. S34 and S35, ESI†).

**Fig. 10:**
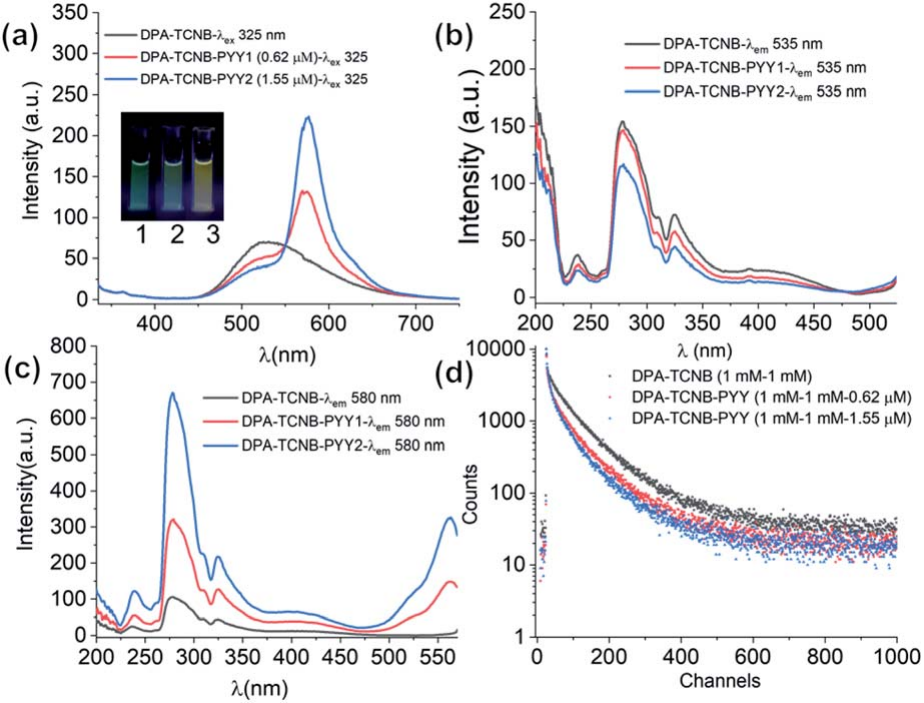
(a) Emission spectra recorded for only DPA–TCNB suspension, and DPA–TCNB–PYY suspensions with increasing dye concentration. Excitation spectra recorded for same samples for (b) CT emission at 535 nm and (c) pyronin Y emission at 580 nm. (d) Lifetime data for 535 nm emission for DPA–TCNB and DPA–TCNB–PYY with 0.62 μM and 1.55 μM PYY.

The fluorescence emission was recorded in solution for the DPA–TCNB–PYY tri-component assembly with increasing pyronin Y concentration. As clearly demonstrated in Fig. 10a, the intensity of CT and pyronin Y emission bands decreased and increased, respectively, upon increasing the concentration of pyronin Y. The excitation spectra were recorded for CT emission at 535 nm upon addition of pyronin Y, which showed a reduction in intensity with increasing pyronin Y concentration as expected (Fig. 10b), demonstrating that the reduction in CT band intensity is due to sensitisation of the pyronin Y moiety. Furthermore, the excitation spectra recorded for pyronin Y emission at 580 nm in the DPA–TCNB–PYY tri-component assembly show (Fig. 10c) a similar excitation band as observed for DPA–TCNB for 535 nm emission, with intensity in the order DPA–TCNB–PYY_1.55 μM_ > DPA–TCNB–PYY_0.62 μM_ > DPA–TCNB, indicating increased energy transfer efficiency with increasing dye concentration.

The excited state decay for CT emission at 535 nm was also recorded at different pyronin Y concentrations. These were best fitted to bi-exponential decay, from which the average lifetimes calculated for the DPA–TCNB bi-component and DPA–TCNB–PYY tri-component assembly with pyronin Y concentrations of 0.62 μM and 1.55 μM were 78.48 ns, 69.75 ns, and 60.77 ns, respectively (Fig. 9d and Table S2, ESI†). A decrease in the excited state lifetime for CT emission at 535 nm was observed in the presence of pyronin Y due to the energy transfer process.

**Fig. 11:**
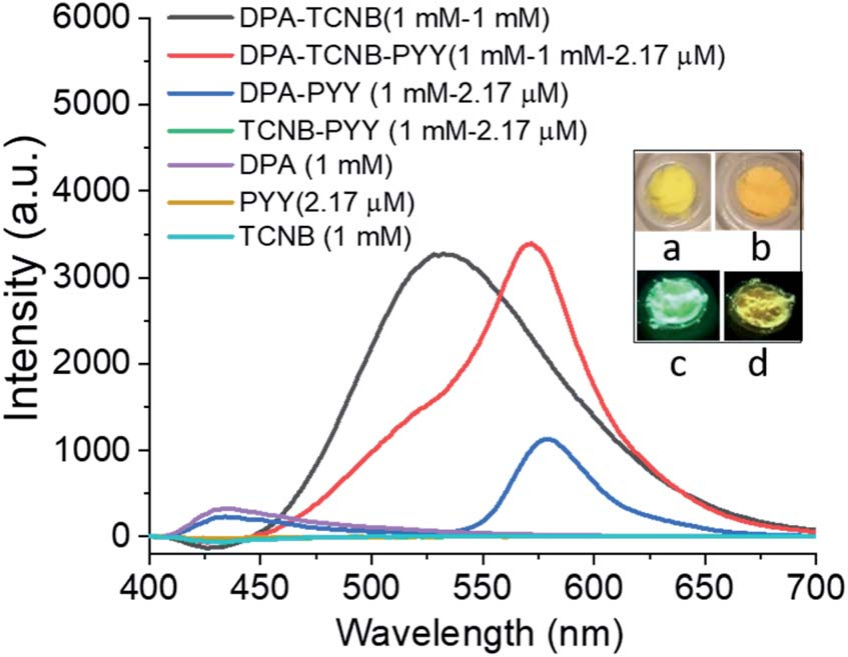
Solid state emission spectra of DPA–TCNB (black line), DPA–TCNB–PYY (red line), DPA–PYY (blue line), TCNB–PYY (green line), DPA (violet line), pyronin Y (yellow line), and TCNB (greenish blue) on 325 nm excitation; inset pictures (a and b) in daylight, (c and d) under a 365 nm UV lamp for DPA–TCNB (a and c) and DPA–TCNB–PYY (b and d), respectively.

The fluorescence solid-state emission spectrum was recorded for the resulting PYY–DPA–TCNB tri-component system (1 mM DPA, 1 mM TCNB, and 2.17 μM pyronin Y), and compared with the solid-state emission spectra of the PYY–DPA and PYY–TCNB samples, as shown in Fig. 11. It is known that pyronin Y, in the solid state, is non-emissive, due to aggregation-induced self-quenching. However, within the PYY–DPA bi-component system, a clear pyronin Y emission band was observed, as demonstrated in Fig. 11, which summarises the results obtained from this investigation. The DPA microsphere matrix stops the fluorescence self-quenching by ensuring ordered assembly and inhibiting self-aggregation. Furthermore, the pyronin Y emission intensity increased significantly in the PYY–DPA–TCNB tri-component assembly, and this was followed by a concomitant decrease in the CT band at 535 nm, demonstrating that sensitisation of the pyronin Y excited state occurs, upon comparison with that observed for the DPA–TCNB assembly under the same experimental conditions. This signifies that the energy transfer from the DPA–TCNB CT complex to pyronin Y in the microsphere matrix is highly effective indicating a ‘stepping stone’ sensitisation of the pyronin Y emitter. This emission was clearly visible to the naked eye and is shown in Fig. 11a–d inset pictures.

### Morphological and confocal fluorescence microscopy examinations of the DPA–TCNB–PYY tri-component system

**Fig. 12:**
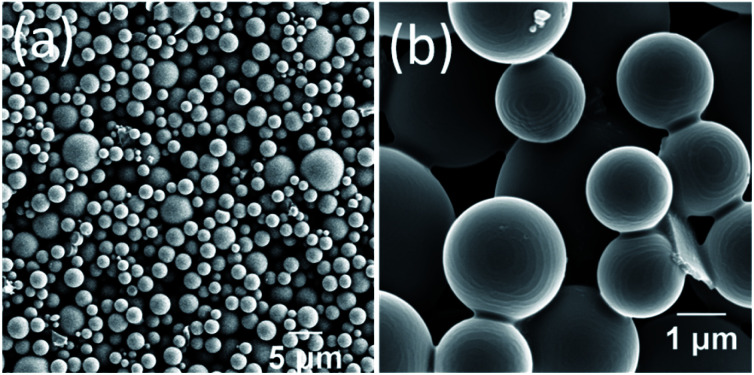
(a and b) SEM images of pyronin Y doped DPA–TCNB microspheres.

Having examined the emission properties of the DPA–TCNB–PYY tri-component system, we next investigated the morphological properties of the assembly. The SEM images recorded for the DPA–TCNB–PYY assembly showed spherical morphology (Fig. 12) and were similar to those of the DPA–TCNB co-assembly. The wide size distribution was observed for the particles with most particles within the ∼1.5–5 μm range.

**Fig. 13:**
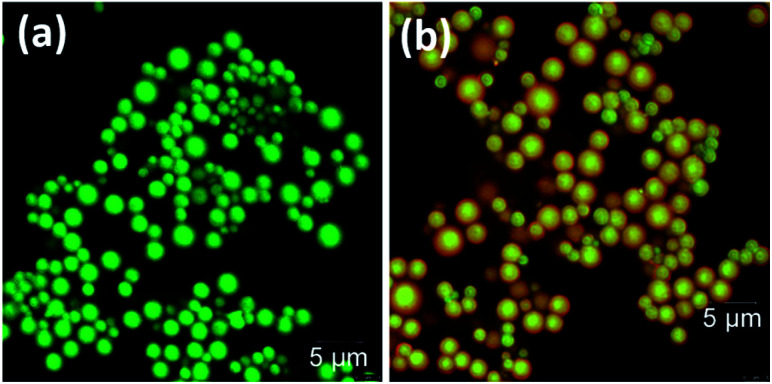
Confocal images of (a) DPA–TCNB and (b) DPA–TCNB–PYY.

Confocal fluorescence images were also recorded for the DPA–TCNB and DPA–TCNB–PYY systems with increasing PYY concentrations. While for DPA–TCNB emission was observed only in the green channel (Fig. 13a and S36a†) as previously demonstrated in Fig. 5, the emission from the DPA–TCNB–PYY assembly was observed both in the green channel and red channel (Fig. 13b and S36b–d†). Moreover, an increase in the emission intensity (*i.e.* in the pyronin Y centred emission) was also seen in the red channel upon increasing pyronin Y concentration, mirroring that seen in solution and the solid state discussed above. The extracted emission spectra from the confocal microscopy scanning for DPA–TCNB and DPA–TCNB–PYY with increasing pyronin Y concentration showed a slight red shift in *λ*_max_ at higher dye concentration (1.78 μM) (Fig. S37†), which is due to the emission band at 575 nm for the pyronin Y dye having higher relative intensity than the CT emission band at 535 nm. Using confocal fluorescence microscopy, 3D images were generated for the DPA–TCNB–PYY (Fig. S38 and S39, ESI†) assembly, which demonstrated the spherical-shaped structure. The more intense CT emission was observed from the centre of the sphere and while red pyronin Y emission was observed uniformly from all over the sphere as shown in Fig. S38 and S39.†

## Conclusions

In conclusion, we have observed the formation of microspheres from a low molecular weight bis-naphthyl dipicolinic amide (DPA) derivative in an ACN/EtOH/water mixture. Furthermore, green fluorescent microspheres were formed by co-assembling DPA with TCNB, which displayed an intensity rise with particle growth as observed by fluorescence and SEM investigations. The solid-state structural analysis of a DPA–TCNB co-crystal confirms the CT interaction between DPA and TCNB. The alternative stacking arrangement was observed in the extended crystal structure, which was characterized by PXRD, and using solid-state emission and excitation measurement. The MO diagram and energy level calculations re-established that the CT complex formation induced green emission from the DPA–TCNB co-assembly. We have observed that the microsphere can act as a matrix for energy transfer, and energy transfer was observed from the green-emitting CT complex to the red-emitting pyronin Y dye in the tri-component co-assembly. This work should motivate researchers to investigate the effect of ligand chirality on CT emission of the co-assembly and other light-harvesting assemblies. Furthermore, it will be intriguing to investigate the formation of fluorescent co-crystals with similar types of molecules.

## Data availability

Crystallographic data for the co-crystal has been deposited at the Cambridge Crystallographic Data Centre (CCDC) under deposition number 2155482. Experimental methods and data and CIF files supporting the findings of this study have been provided as ESI.†

## Author contributions

T. G. (Thorfinnur Gunnlaugsson) and TG (Tumpa Gorai) conceived the idea. TG synthesized the material, performed spectroscopy and SEM experiments, and analysed the data. JL performed single-crystal and powder X-ray diffraction and structure determination. DU performed the DFT calculation. GM and TG performed all confocal microscopy experiments. T. G., TG, JL, and DU wrote the manuscript.

## Conflicts of interest

There are no conflicts to declare.

## Supplementary Material

SC-013-D2SC02097A-s001

SC-013-D2SC02097A-s002
