# Supplementary material for: Hierarchical supramolecular co-assembly formation employing multi-component light-harvesting charge transfer interactions giving rise to long-wavelength emitting luminescent microspheres
Source: Chem Sci. 2022 May 31;13(26):7805–13. doi: 10.1039/d2sc02097a (PMC9258320; doi:10.1039/d2sc02097a)
Supplement: SC-013-D2SC02097A-s001 [file SC-013-D2SC02097A-s001.pdf]

Electronic Supplementary Material (ESI) for Chemical Science.
This journal is © The Royal Society of Chemistry 2022

Supporting information

Hierarchical supramolecular co-assembly formation employing multi-component light-harvesting charge transfer interactions giving rise to long-wavelength emitting luminescent microspheres

Tumpa Gorai,*[a] June I Lovitt ,[a,b] Deivasigamani Umadevi,[c] Gavin McManus,[d] and Thorfinnur Gunnlaugsson*[a,b,e]

[a]*School of Chemistry and Trinity Biomedical Sciences Institute (TBSI), Trinity College Dublin, The University of Dublin, Dublin 2, Ireland. E-mail: gorait@tcd.ie, gunnlaut@tcd.ie.*

[b]*Advanced Materials and BioEngineering Research (AMBER) Centre, Trinity College Dublin, The University of Dublin, Dublin 2, Ireland.*

[c]*Department of Chemistry, Indian Institute of Technology Palakkad (IITPKD), Palakkad-678557, Kerala, India*

[d] *School of Biochemistry and Immunology, Trinity Biomedical Sciences Institute (TBSI), Trinity College Dublin, The University of Dublin, Dublin 2, Ireland.*

[e]*Synthesis and Solid State Pharmaceutical Centre (SSPC), Ireland*

Contents

1. Materials and methods 3

2. Experimental for X-ray crystallography 3

3. DFT calculation 4

4. Synthesis of 4-hydroxy-N^2,N^6-bis((R)-1-(naphthalen-2-yl)ethyl)pyridine-2,6-dicarboxamide (DPA) derivative 4

5. Microsphere preparation procedure 6

6. Sample preparation procedure for solid and suspension state emission, solid state absorption, SEM, and confocal microscopic study 7

7. SEM images of DPA spheres in ACN/EtOH/water (8/32/60%) immediately after preparation 7

8. SEM images of DPA spheres in ACN/EtOH/water (8/32/60%) after 24h aging 8

9. SEM images of DPA in ACN/EtOH (20/80%) 8

10. SEM images of DPA in ACN/EtOH/H_2O (12/48/40%) 9

11. Absorption spectra for DPA solution on serial dilution in ACN/EtOH/H_2O (10/40/50%) 10

12. Picture of DPA-TCNB with increasing water percentage (0-50%) 12

13. Comparison of absorption spectra of DPA, TCNB, and DPA-TCNB at high and low concentrations 12

14. Absorption, emission, and excitation spectra of DPA-TCNB with increasing water percentage 13

15. Comparison of emission spectra of DPA, TCNB and DPA+TCNB 14

16. Absorption, emission, and excitation study of DPA in ACN/EtOH/water mixture with increasing water percentage 15

17. Comparison of excitation spectra and absorption spectra of DPA+TCNB 16

18. Emission spectra of DPA-TCNB suspension with variable TCNB concentration 17

19. Excitation spectra of DPA-TCNB suspension with variable TCNB concentration 18

20. Emission spectra of DPA-TCNB suspension ACN/EtOH/water (8:32:60%) on cooling from 50 °C to 22 °C 18

21. SEM images of DPA-TCNB solution in ACN/EtOH (20/80%) 19

22. SEM images of DPA-TCNB suspension in ACN/EtOH/water (8:32:60%) 19

23. Emission of DPA-TCNB suspension in ACN/EtOH/water (8:32:60%) recorded with time 20

24. SEM and Confocal microscopy images of TCNB... 21

25. Table S1 Crystal data and structure refinement for DPA-TCNB co-crystal. 22

26. Single crystal and calculated Hirschfield surface of DPA-TCNB co-crystal............................... 23

27. Thermogravimetric analysis:.. 24

28. NMR spectral comparison.. 25

29. Infrared spectral comparison.. 25

30. Spectral overlap between emission spectra of DPA-TCNB CT co-assembly and Pyronin Y absorption 26

31. Comparison of emission and excitation spectra of Pyronin Y 26

32. Table S2: Lifetime data of CT emission at 535 nm 28

33. Confocal images of DPA-TCNB and DPA-TCNB-PYY 29

34. Confocal image of DPA+TCNB microsphere in 3D mode 30

35. References 30

1. Materials and methods

Chelidamic acid monohydrate, (*R*)-(+)-1-(1-Naphthyl)ethylamine, tetracyanobenzene (TCNB) were purchased from TCI Europe. 1-hydroxybenzotriazole hydrate (HOBt), di-isopropyl ethyl amine (DIPEA), N-(3-Dimethylaminopropyl)-N′-ethylcarbodiimide hydrochloride (EDC) were purchased from Sigma Aldrich. Pyronine Y was from ACROS organic. Water purified in Millipore Milli Q water purification system was used for all experiments. Spectroscopic and HPLC grade solvents were used for all experiments. NMR spectra were recorded in a Bruker Spectrospin DPX-400 instrument operating at 400 MHz for ^{1}H and 100 MHz for ^{13}C NMR or a Bruker AV-600 instrument operating at 600 MHz for ^{1}H NMR and 150 MHz for ^{13}C NMR. IR spectra were recorded in a PerkinElmer Spectrum One FTIR spectrometer within 4000-650 cm^{-1}. Mass spectra were recorded in Micro mass time of flight mass spectrometer (tof), interfaced to a Waters 2690 HPLC. Elemental analysis was performed on Exeter Analytical CE440 elemental analyser at the microanalysis laboratory, School of Chemistry and Chemical Biology, University College Dublin. Thermogravimetric analysis for powder and crystalline charge transfer complexes were performed on Perkin Elmer Pyrus 1 TGA equipped with an ultra-micro balance having sensitivity of 0.1 microgram within temperature range of 30 - 600 °C with a scan rate 5 °C min^{-1}, under a N_2 gas flow of 20 mL/min. Absorption spectra of solution was recorded with 1 cm path length quartz cuvette in Cary 60 UV-Vis. Emission spectra of solutions and suspensions were recorded with 1 cm path length quartz cuvette in Cary eclipse fluorescence spectrophotometer. Solid state UV Spectra were recorded with solid sample put in between quartz slides and recorded in Perkin Elmer Lambda 650 UV/Vis spectrophotometer with 150 mm integrating sphere. For solid state emission spectra were recorded by drop-casting sample in 96 well plate and dried sample was recorded in SpectraMax M3 Microplate Reader. Lifetime measurement was done in Horiba fluorescence spectrometer with 340 nm laser excitation. Scanning Electron Microscopy (SEM) was carried out on Carl Zeiss Ultra SEM. The samples were coated with a thin layer of gold-palladium using a sputtering procedure. Diffraction data were collected using a Bruker D8 Quest ECO instrument using graphite-monochromated Mo Kα (λ = 0.71073 Å) radiation. X-ray powder diffraction patterns were collected using a Bruker D2 Phaser instrument with Cu Kα radiation (λ = 1.5418 Å). Samples were ground and mounted on silicon sample holders, and data were collected in the 2θ range 5 – 55° at room temperature. The patterns were compared with those simulated from the single-crystal data collected at 100 K.

2. Experimental for X-ray crystallography

Diffraction data were collected using a Bruker D8 Quest ECO instrument using graphite-monochromated Mo Kα (λ = 0.71073 Å) radiation. Datasets were collected using ω and ϕ scans with the samples immersed in oil and maintained at a constant temperature of 100 K using a Cobra cryostream. The reflection data were reduced and processed using the Bruker APEX-3 suite of programs.[1] Multi-scan absorption corrections were applied using SADABS.[2] The diffraction data were solved using direct methods with SHELXT and refined by full-matrix least-squares procedures with SHELXL-2015 within the OLEX-2 GUI.[3-4] All non-hydrogen atoms were refined with anisotropic displacement parameters. All carbon-bound hydrogen atoms were placed in calculated positions and refined with a riding model, with isotropic displacement parameters equal to either 1.2 or 1.5 times the isotropic equivalent of their carrier atoms. Where appropriate, the positions of the hydrogen atoms involved in hydrogen bonding interactions were refined to provide the best fit for the residual Fourier peaks and assigned a U_{iso} value equal to 1.5 times that of the nearest associated atom, with the appreciation that the exact positions of these atoms cannot be meaningfully inferred from X-ray diffraction data. Specific refinement strategies are outlined in the crystallographic information file. CCDC 2155482.

3. DFT calculation

Theoretical calculations were done by employing density functional theory (DFT) to understand the mechanism of charge transfer between 1:2 complexes of DPA and TCNB. All the possible orientations of the DPA+2TCNB complex were considered and only the minimum energy structure was reported. All the calculations were carried out by using Gaussian 16 package.[5] Geometry optimisations were performed using the B3LYP hybrid functional with the DFT-D3 dispersion corrections and the 6-31G(d,p) basis set. The energy values were fine-tuned by single point calculations by the same functional using 6-311++G(d,p) basis set.

4. Synthesis of 4-hydroxy-N^2,N^6-bis((R)-1-(naphthalen-2-yl)ethyl)pyridine-2,6-dicarboxamide (DPA) derivative

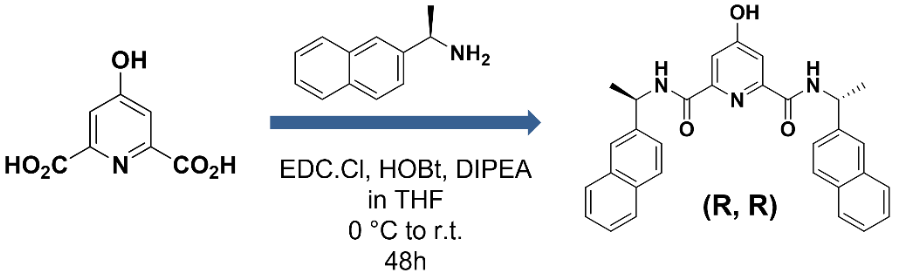

Scheme S 1: DPA synthetic scheme

DPA(R, R) was synthesized following earlier reports.[6] 1-ethyl-3-(3-dimethylaminopropyl)carbodiimide hydrochloride (EDC, 2.3g, 11.9 mmol), 1-hydroxybenzotriazole hydrate (HOBt hydrate, 1.62g, 11.9 mmol) and N, N-Diisopropylethylamine (DIPEA, 4.75mL, 27.3 mmol) were added to a solution of Chelidamic acid monohydrate (1g, 4.97 mmol) in anhydrous tetrahydrofuran (50 mL) at 0°C and stirred for 30 min. Later, (R)-1-(2-naphthyl) ethylamine (2g, 11.6 mmol) was added and stirred at room temperature for 48h. THF was removed, the compound was re-dissolved in dichloromethane, washed with 0.1N HCl (50 mL), aqueous sat. Sodium bicarbonate (30 mL) and brine (30 mL). The organic layer was dried over Na_2SO_4, and after removal of solvent under vacuum, the crude was purified by flash silica column (24g, 0-5% MeOH in DCM) to afford DPA (R, R) as off white solid (1.3g, yield 55%). MP 123-125 °C, ^{1}H NMR (600 MHz, DMSO-D_6) δ : 11.43 (s, 1H), 9.41 (d, *J* = 8.4 Hz, 2H), 7.92-7.88 (m, 8H), 7.63 (dd, *J* = 8.5 Hz, 1.7 Hz , 2H), 7.59 (d, *J* = 1.9 Hz, 2H), 7.52-7.46 (m, 4H), 5.42 (p, *J* = 7.1 Hz, 2H), 1.70 (d, *J* = 7.0 Hz, 6 H); ^{13}C NMR(150 MHz, DMSO-D_6) δ ppm : 166.46, 162.93, 151.10, 141.49, 132.82, 132.06, 127.94, 127.65, 127.45, 126.19, 125.75, 125.09, 124.10, 111.86, 48.31, 21.72: IR v_{max}(cm^{-1}): 3287, 3052, 2975, 2932, 1746, 1650, 1602, 1518, 1437, 1358,1318 1235, 1179, 1132, 1102, 1041, 955, 889, 856, 820 746, 695;HRMS (m/z) calculated for $C_{31}H_{27}N_3NaO_3$ [M + Na] 512.1950, observed for $C_{31}H_{27}N_3NaO_3$ [M + Na] 512.1942; elemental analysis calculated for $C_{31}H_{27}N_3O_3$. 0.5H_2O, C 74.68, H 5.66, N 8.43, observed C 74.76, H 5.50, O 8.34

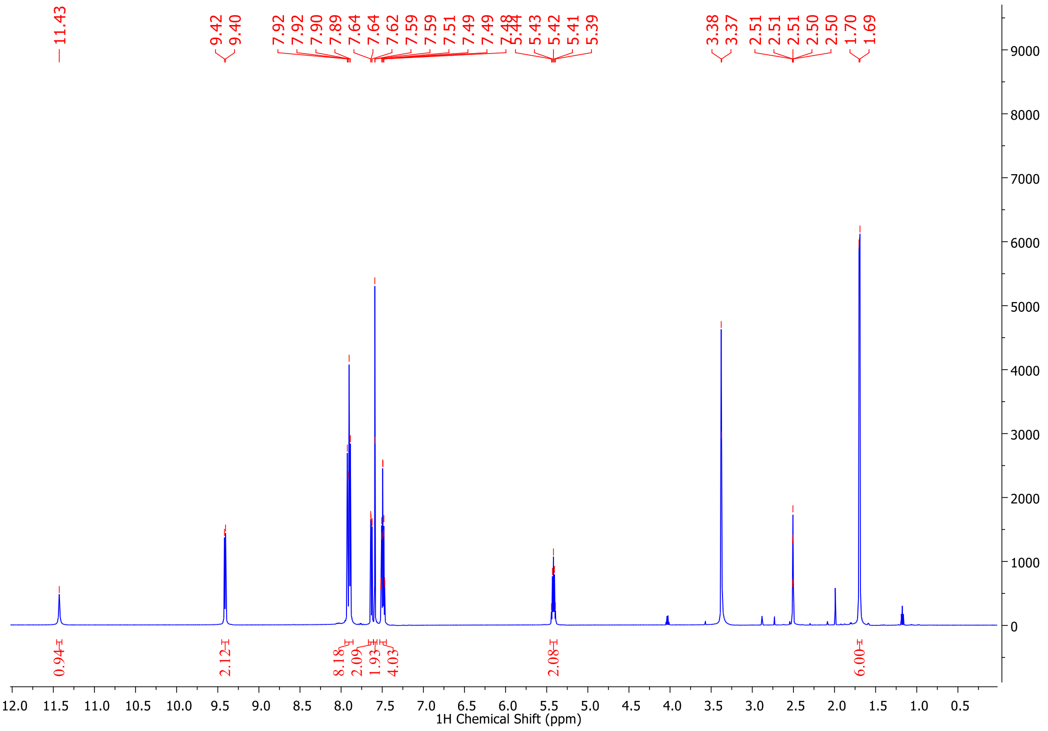

Figure S 1: ^{1}H NMR of DPA in DMSO-D$_6$

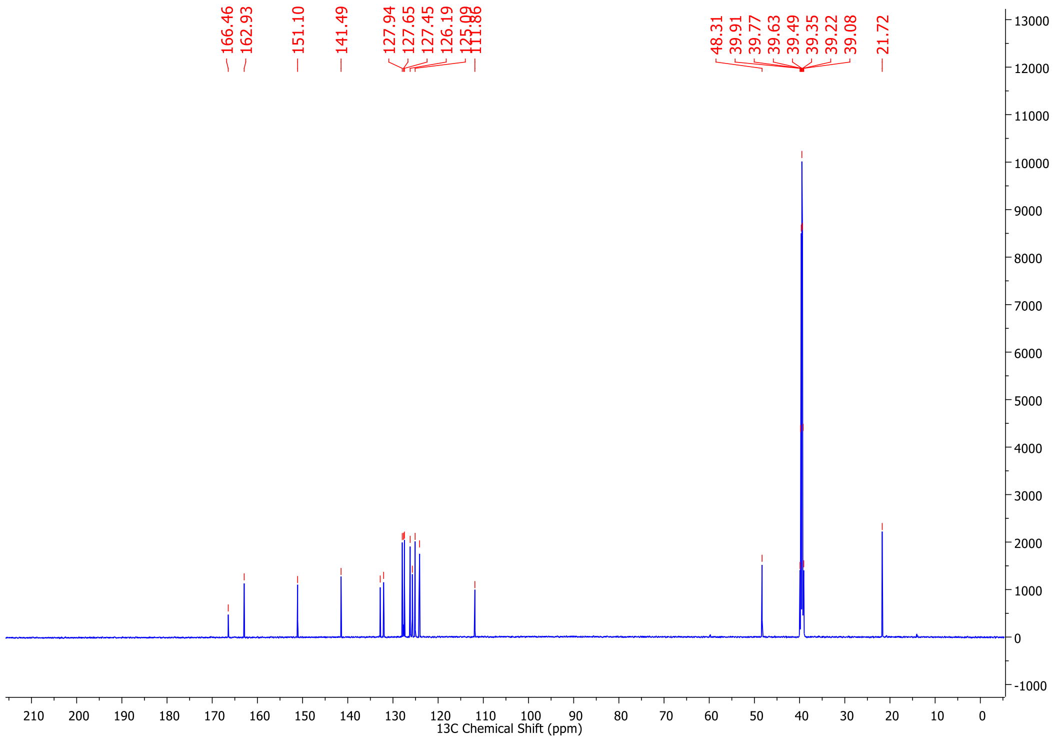

Figure S 2: ^{13}C NMR of DPA in DMSO-D$_6$

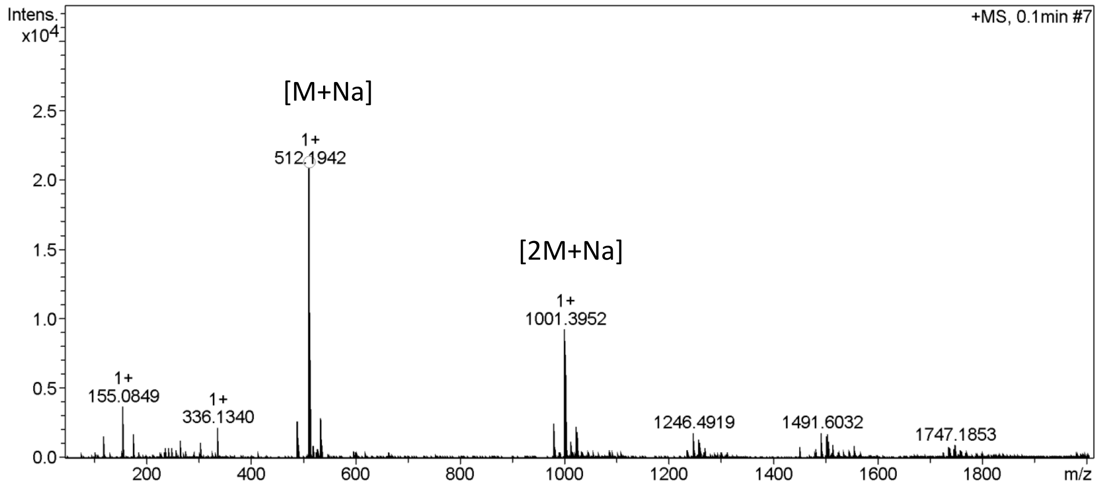

Meas. m/z	#	Ion Formula	m/z	err [mDa]	err [ppm]	rdb	N-Rule	e⁻ Conf	mSigma
512.194184	1	C31H27N3NaO3	512.194462	-0.3	-0.5	19.5	ok	even	6.3

Figure S 3: The HRMS spectrum of DPA

5. Microsphere preparation procedure

For the preparation of microsphere, DPA was dissolved (2.44 mg) in ACN/EtOH (20/80%) (2 ml) solvent mixture. Following that water (3 mL) was added and a homogeneous suspension formed. On aging for 24h, the suspension settled down in the bottom of a container. The suspension was re-dispersed on sonication (Figure S3) and studied for spectroscopic, and microscopic analysis.

For preparation of DPA-TCNB bi-component microsphere, DPA solution (1 mL, 5 mM) in ACN/EtOH (20/80%) was mixed with TCNB solution (1 mL, 5 mM) in ACN/EtOH (20/80), and water (3 mL) was added to prepare a suspension in ACN/EtOH/water (8/32/60%) mixture with final DPA and TCNB concentrations 1 mM each in the mixture. The suspension was kept at room temperature for aging.

For the preparation of DPA-TCNB-PYY tri-component microsphere, DPA solution (1 mL, 5 mM) in ACN/EtOH (20/80) was mixed with TCNB solution (1 mL, 5 mM) in ACN/EtOH (20/80), and Pyronin Y solution (310 µM, 3-25 µL) in EtOH. Water (3 mL) was added to prepare a suspension in ACN/EtOH/water (8/32/60%) mixture with final concentration of DPA and TCNB 1 mM each in the mixture and Pyronin Y ≤ 2 µM. The suspension was kept at room temperature for aging.

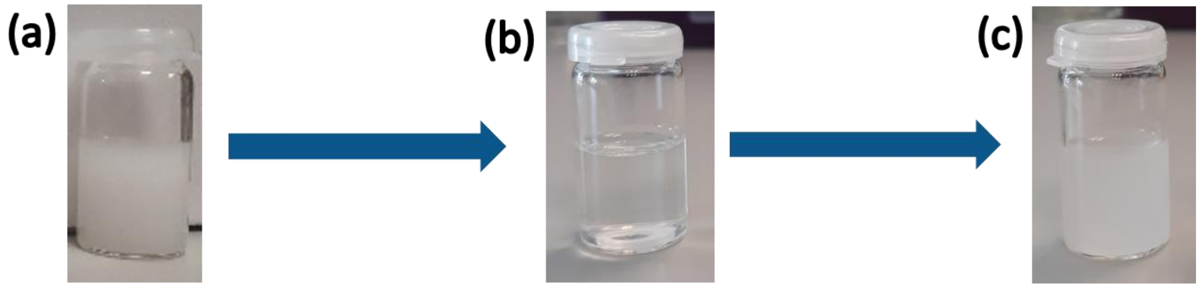

Figure S 4: (a) Picture of DPA suspension immediately after preparation in ACN/EtOH/water (8/32/60%), (b) suspension deposited in the bottom on overnight aging, (c) suspension re-dispersed on sonication.

6. Sample preparation procedure for solid and suspension state emission, solid state absorption, SEM, and confocal microscopic study

The particle deposited at the bottom of vial after 24h aging was re-dispersed as uniform suspension by sonication for ~5 min, the sample was diluted three times with water and the emission and lifetime measurement were done in 1 cm path length quartz cuvette.

For solid state emission measurement, sonicated and re-dispersed sample (20-50 μL) was dropcasted in 96 well plate or dropcasted on quartz slides and air dried.

For solid state absorption measurement, the powder sample was fixed in between two quartz slides and absorption was recorded.

For SEM imaging, the suspension (8 μL) was dropcated on silicon wafer, dried in the air, following that in high vacuum for 1h. For confocal imaging, the sample (20 μL) was dropcasted on 8 or 18 well glass slides and air-dried before measurement.

7. SEM images of DPA spheres in ACN/EtOH/water (8/32/60%) immediately after preparation

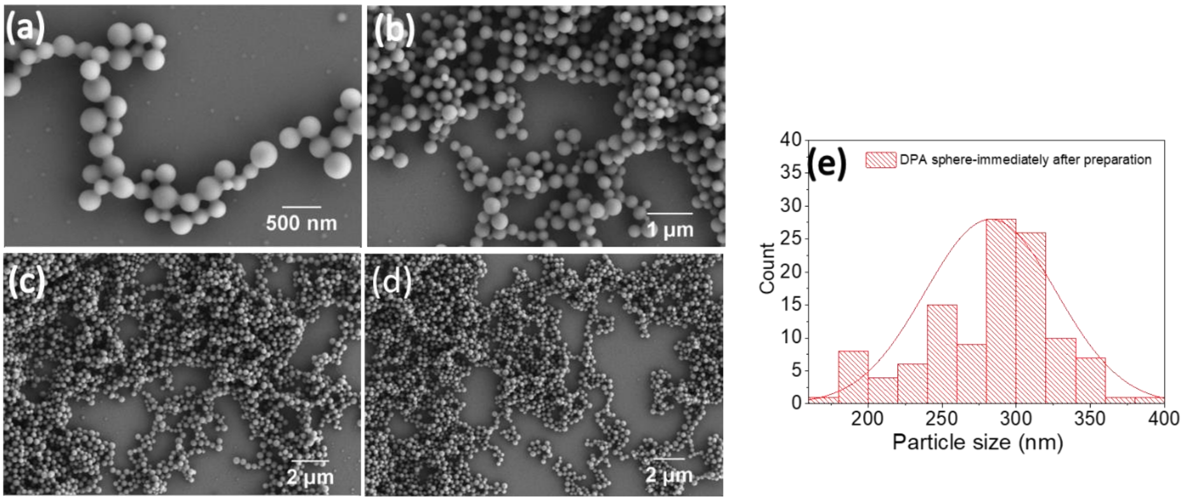

Figure S 5: (a-d) SEM images DPA spheres drop-casted immediately after preparation in ACN/EtOH/water (8/32/60%) composition, (e) histogram plot for particle size distribution.

8. SEM images of DPA spheres in ACN/EtOH/water (8/32/60%) after 24h aging

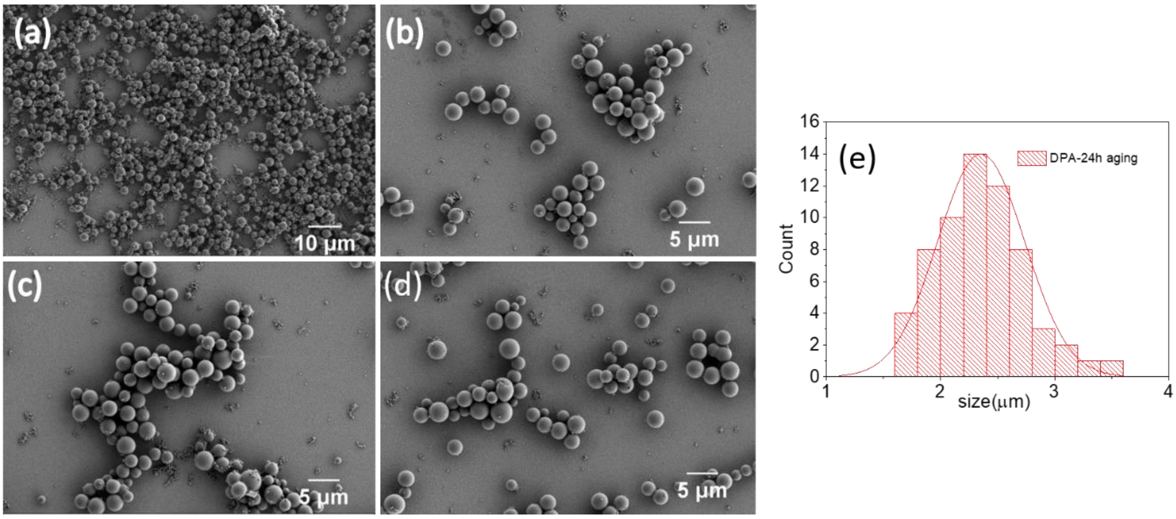

Figure S 6: (a-d) SEM images DPA spheres drop-casted 24h after preparation in ACN/EtOH/water (8/32/60%) composition, (e) histogram plot for particle size distribution.

9. SEM images of DPA in ACN/EtOH (20/80%)

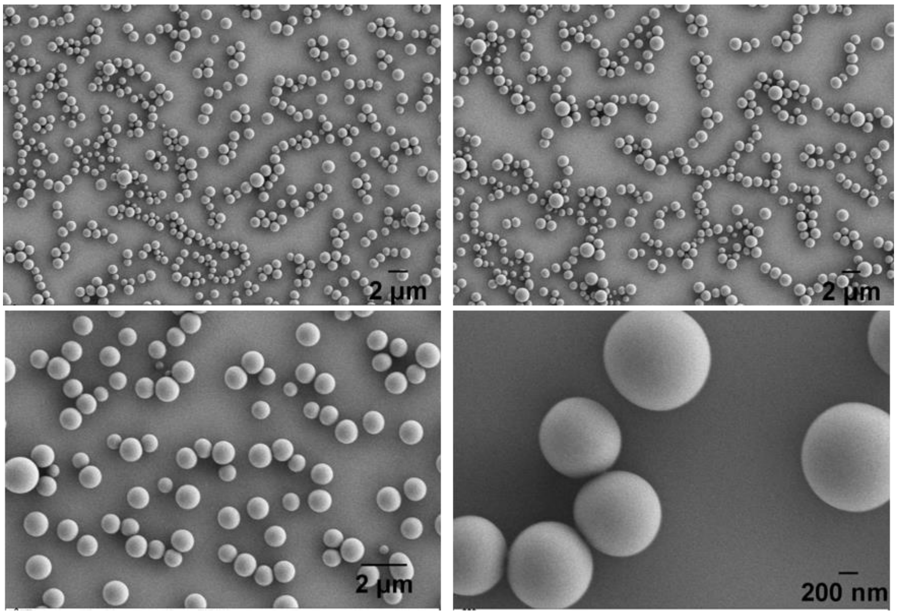

Figure S 7: SEM images for clear solution of DPA sphere in ACN/EtOH (20/80%).

10. SEM images of DPA in ACN/EtOH/H_2O (12/48/40%)

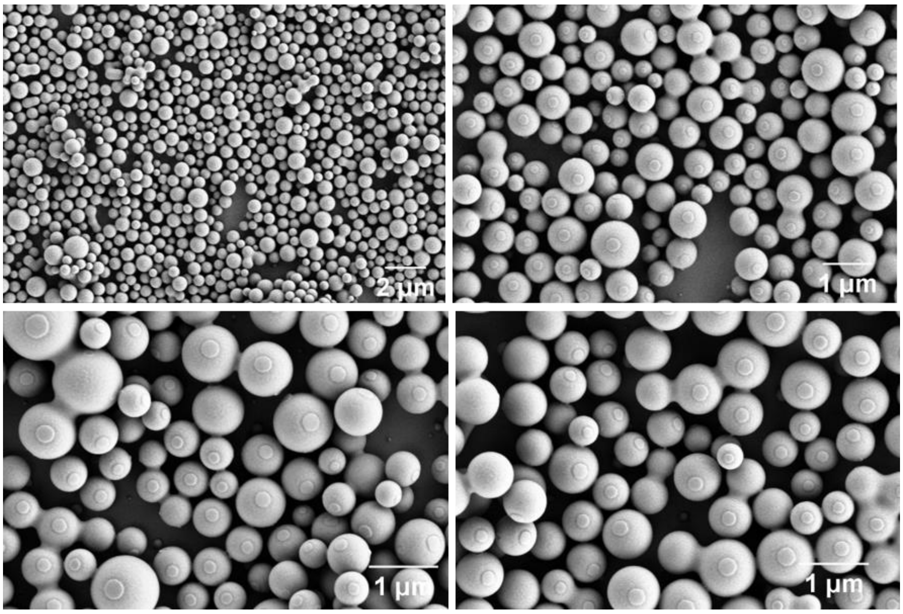

Figure S 8: SEM images for clear solution of DPA sphere in ACN/EtOH/H_2O (12/48/40%).

11. Absorption spectra for DPA solution on serial dilution in ACN/EtOH/H_2O (10/40/50%)

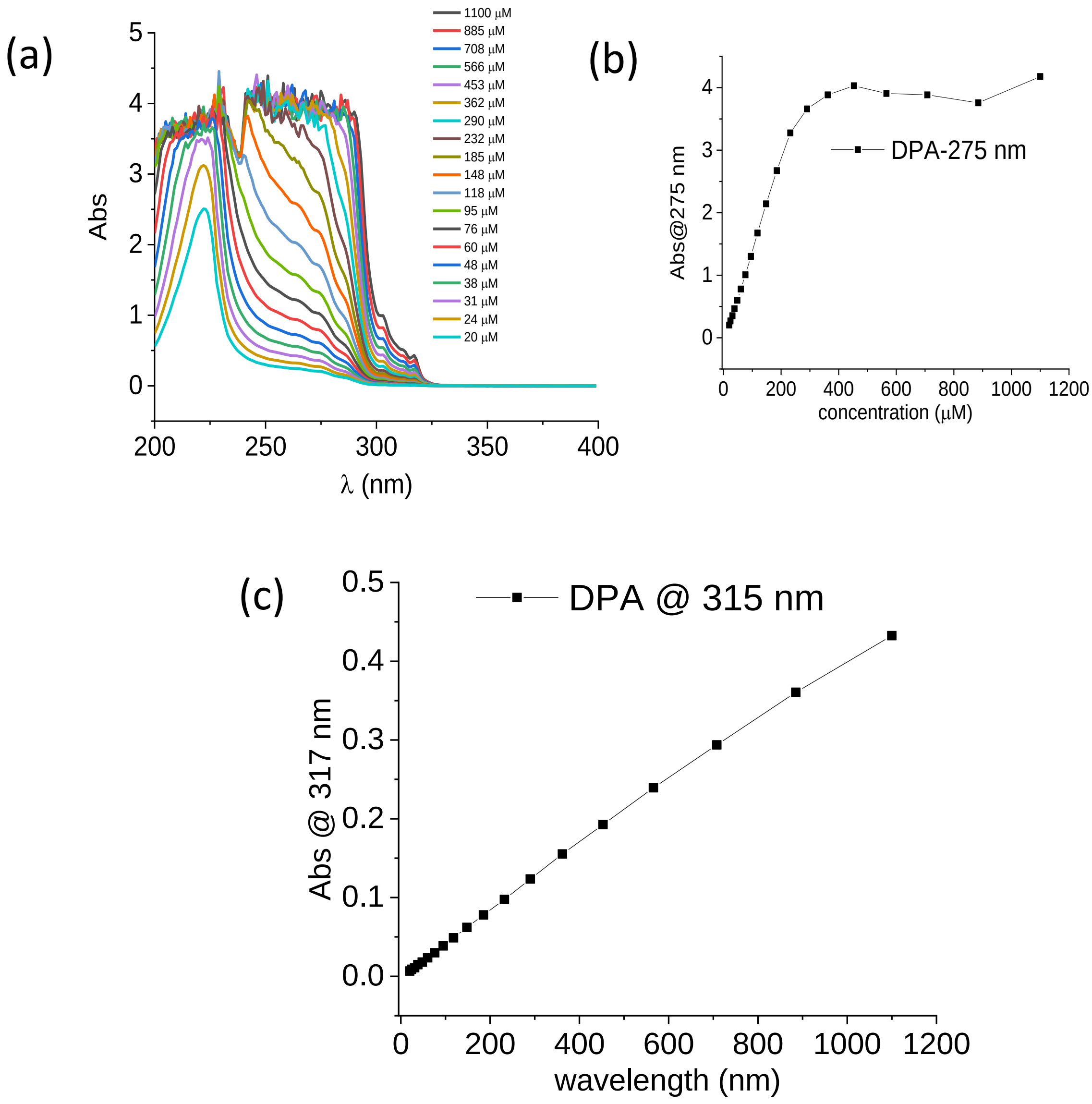

Figure S 9: (a) UV-Vis absorption spectra recorded for DPA (1.1 mM) solution on serial dilution in ACN/EtOH/H_2O (10/40/50%), (b) absorption at 275 nm and (c) absorption at 315 nm plotted with increasing concentration (20-1100 µM).

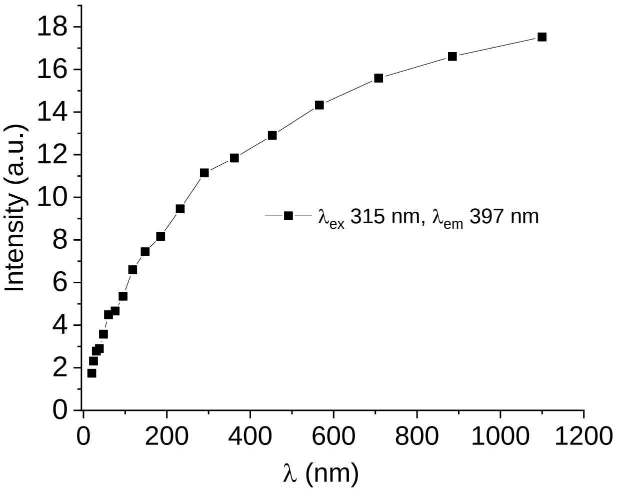

Figure S 10: Emission at 397 (on excitation at 315 nm) with increasing DPA concentrations (20-1100 µM).

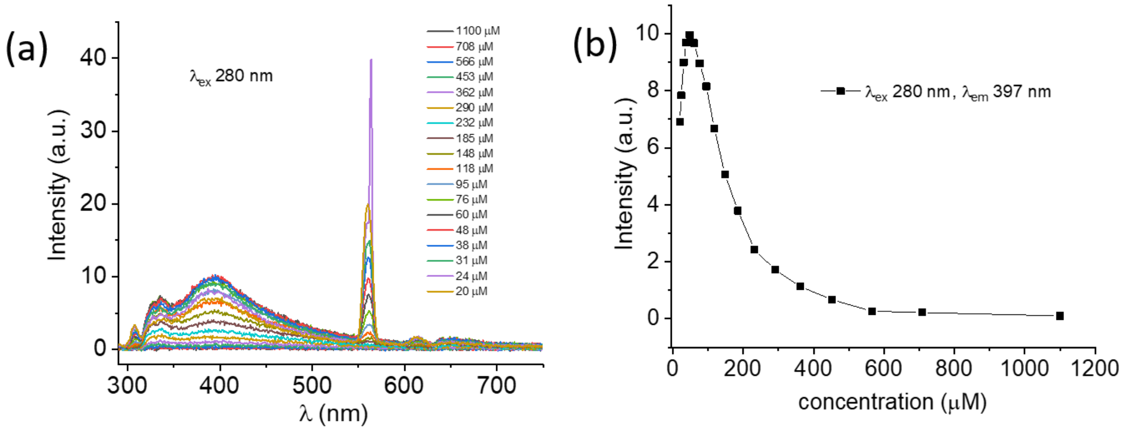

Figure S 11: (a) Emission spectra of DPA in ACN/EtOH/H_2O (10/40/50-%) on increasing concentration on excitation at 280 nm, (b) Emission at 397(on excitation at 280 nm) plotted with increasing DPA concentrations (20-1100 µM).

12. Picture of DPA-TCNB with increasing water percentage (0-50%)

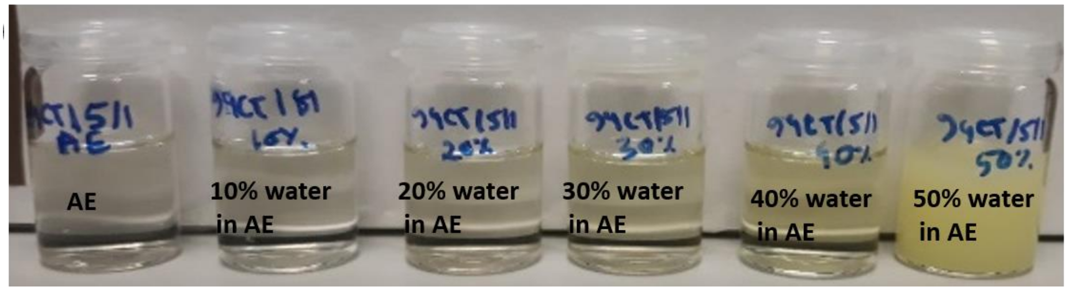

Figure S 12: DPA-TCNB (5 mM each) with increasing water percentage (Since substrate concentration is higher, a lower percentage of water generates suspension).

13. Comparison of absorption spectra of DPA, TCNB, and DPA-TCNB at high and low concentrations

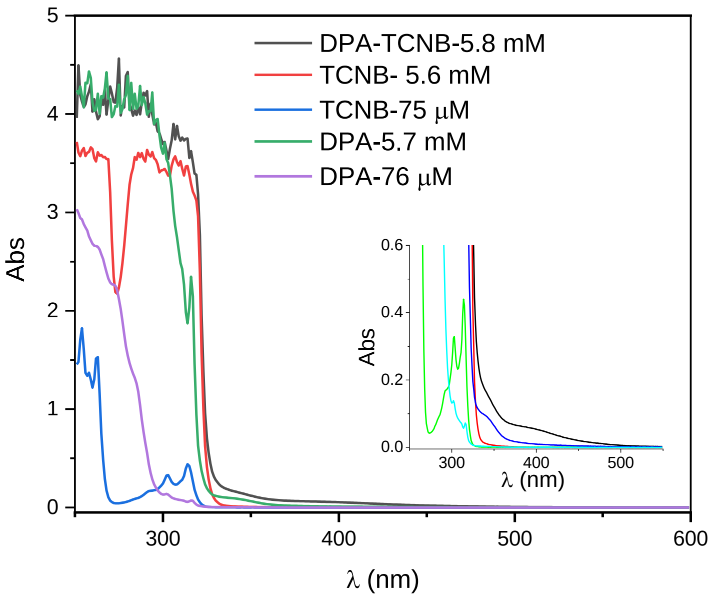

Figure S 13: Absorption spectra of DPA (76 μM, 5.7 mM), TCNB (75 μM, 5.6 μM), DPA-TCNB (5.8 mM) in ACN/EtOH/H_2O (12/48/40%) mixture.

14. Absorption, emission, and excitation spectra of DPA-TCNB with increasing water percentage

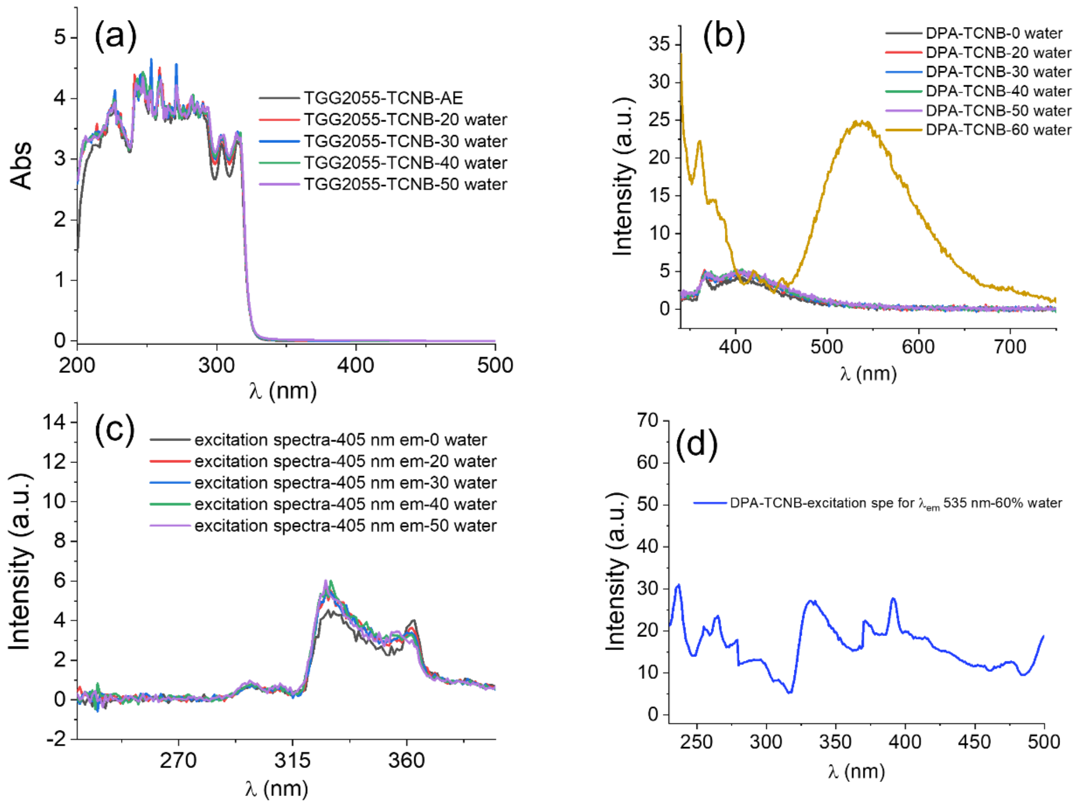

Figure S 14: (a) UV-Vis absorption spectra of DPA + TCNB mixture (1 mM each) with increasing water percentage (absorption spectra was not recorded at 60% water due to suspension formation), (b) Fluorescence emission spectra of DPA + TCNB mixture (1 mM each) with increasing water percentage, λ_{ex} 330 nm, (c) excitation spectra of DPA + TCNB mixture (1 mM each) in 30-50% water for 405 nm emission. (d) excitation spectra of DPA + TCNB mixture (1 mM each) in 60% water for 535 nm emission.

15. Comparison of emission spectra of DPA, TCNB and DPA+TCNB

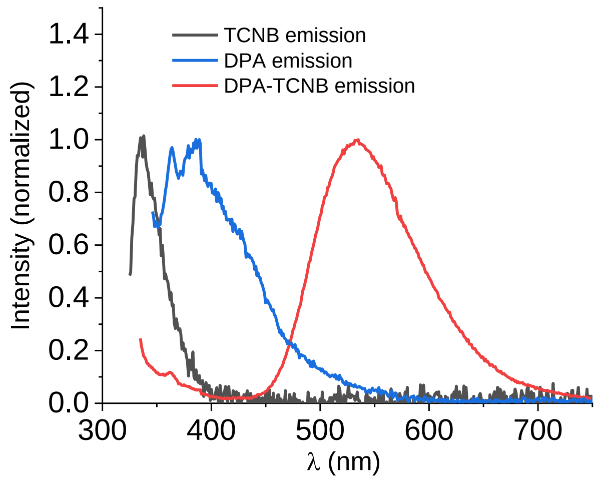

Figure S 15: Emission spectra of TCNB on 315 nm excitation, DPA on 325 nm excitation and DPA-TCNB on 325 nm excitation.

16. Absorption, emission, and excitation study of DPA in ACN/EtOH/water mixture with increasing water percentage

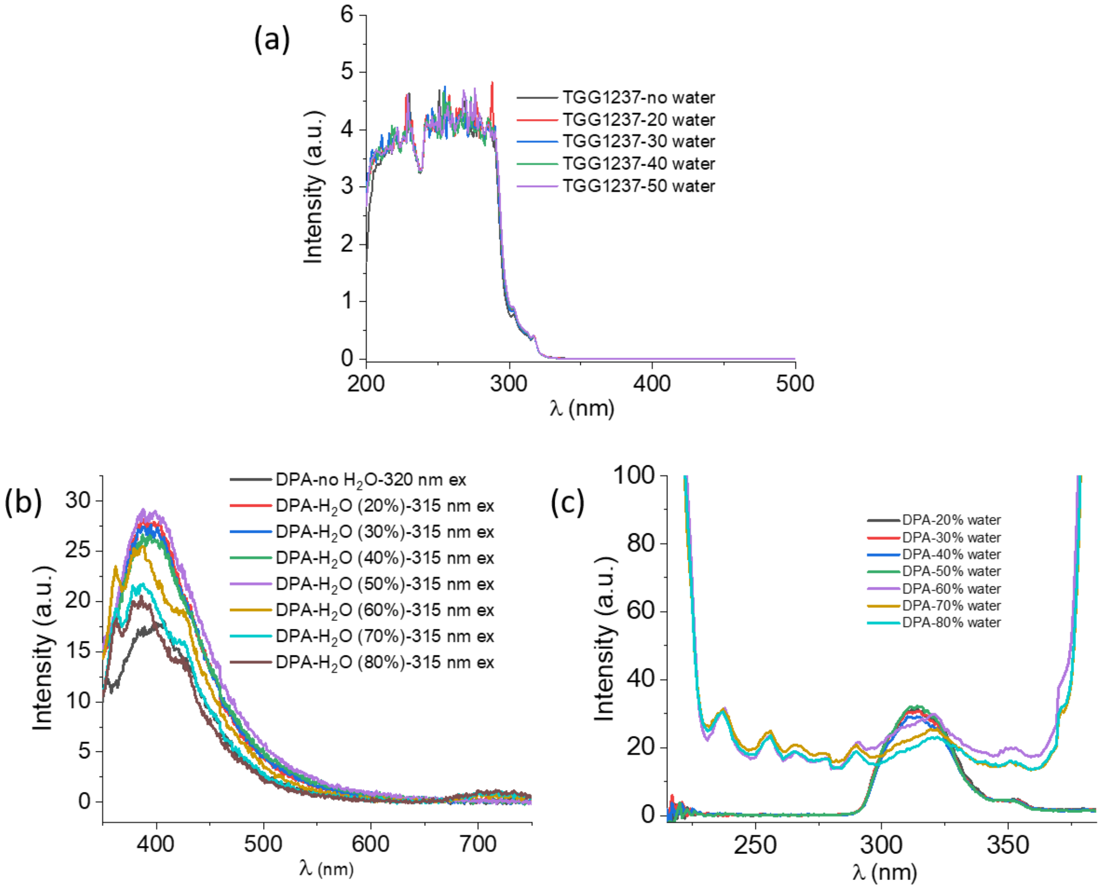

Figure S 16: (a) UV-Vis absorption of DPA sphere solution (1 mM) in water/ACN/EtOH mixture with increasing the percentage of water (absorption were not recorded above 60%, due to formation of suspension), (b) The Fluorescence emission (on excitation at 315 nm) and excitation spectra(for emission at 395 nm) of DPA (1 mM) in ACN/ethanol/water mixture on increasing the percentage of water.

17. Comparison of excitation spectra and absorption spectra of DPA+TCNB

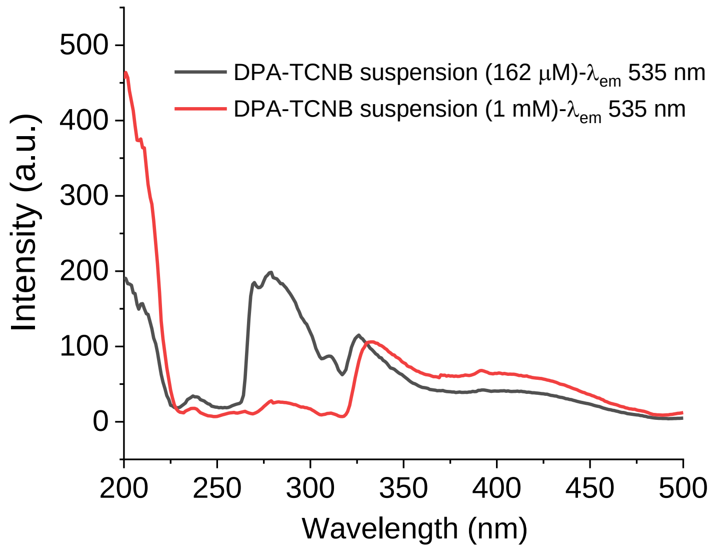

Figure S 17: Comparison of excitation spectra for concentrated (1 mM) and diluted (162 µM) DPA-TCNB suspension.

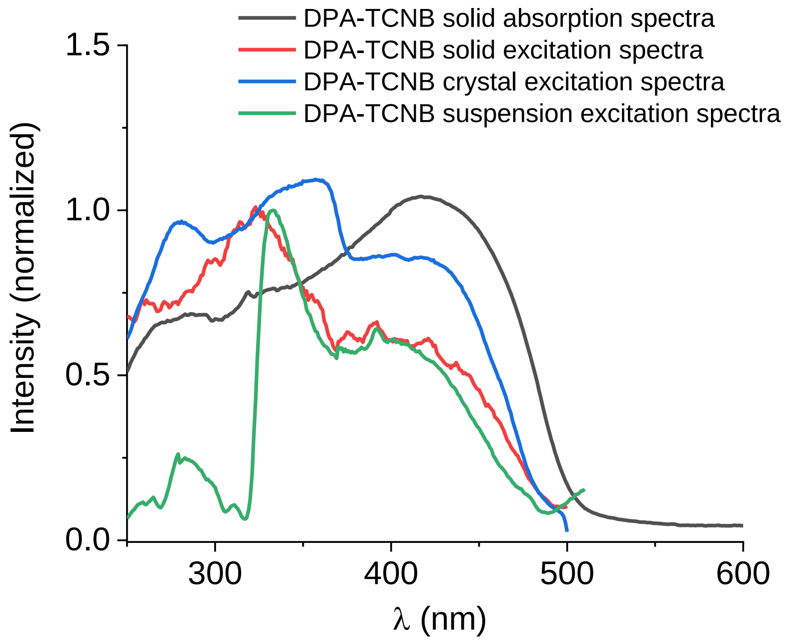

Figure S 18: Comparison of excitation spectra of DPA+TCNB suspension (green line), solid (red line), and crystal (blue line) with DPA+TCNB solid state absorption (black line).

18. Emission spectra of DPA-TCNB suspension with variable TCNB concentration

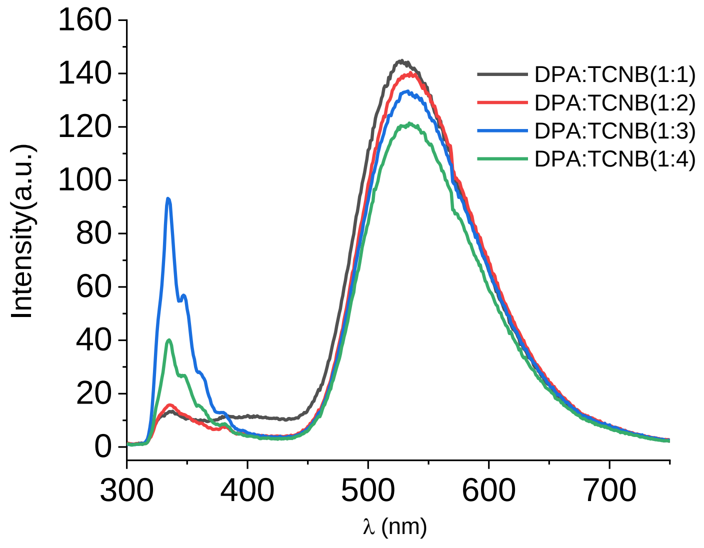

Figure S 19: Emission spectra for DPA-TCNB with DPA (1 mM) and varying TCNB (1-4 mM) on excitation λ_{ex} 280 nm.

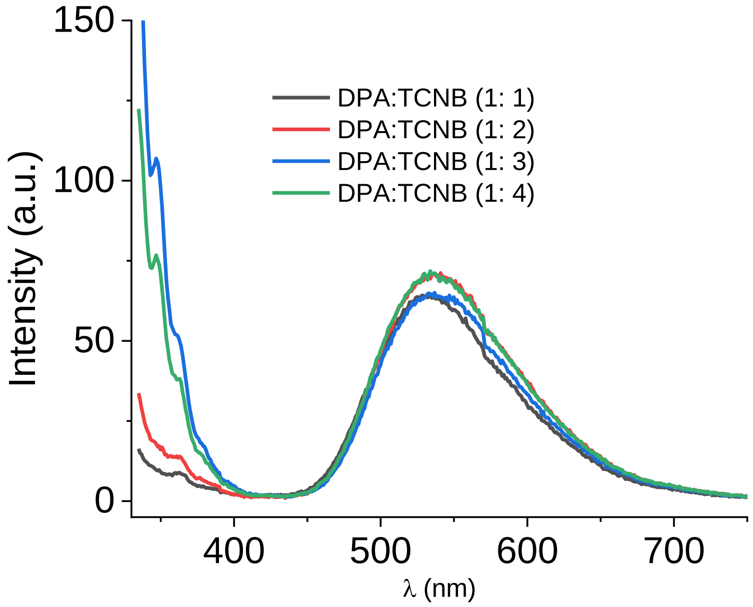

Figure S 20: Emission spectra for DPA-TCNB with DPA (1 mM) and varying TCNB (1-4 mM) on excitation λ_{ex} 325 nm.

19. Excitation spectra of DPA-TCNB suspension with variable TCNB concentration

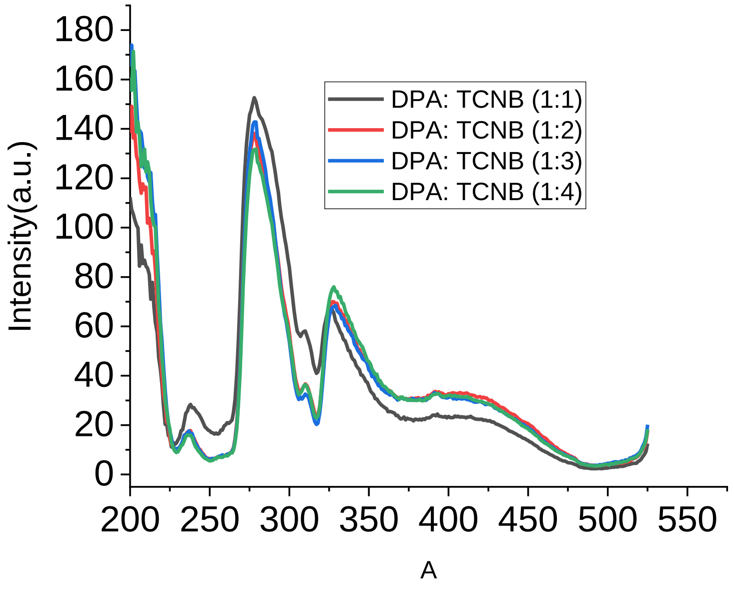

Figure S 21: Excitation spectra for DPA-TCNB with DPA (1 mM) and varying TCNB (1-4 mM) for emission at λ_{em} 535 nm.

20. Emission spectra of DPA-TCNB suspension ACN/EtOH/water (8:32:60%) on cooling from 50 °C to 22 °C

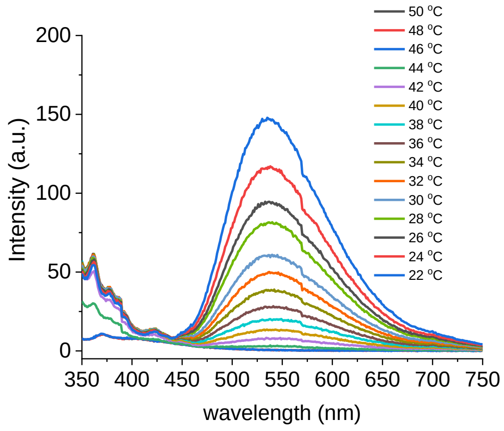

Figure S 22: Emission spectra recorded on λ_{ex} 330 nm for DPA-TCNB suspension(1 mM each) in ACN/EtOH/water (8/32/60%) on gradual cooling from 50 °C to 22 °C.

21. SEM images of DPA-TCNB solution in ACN/EtOH (20/80%)

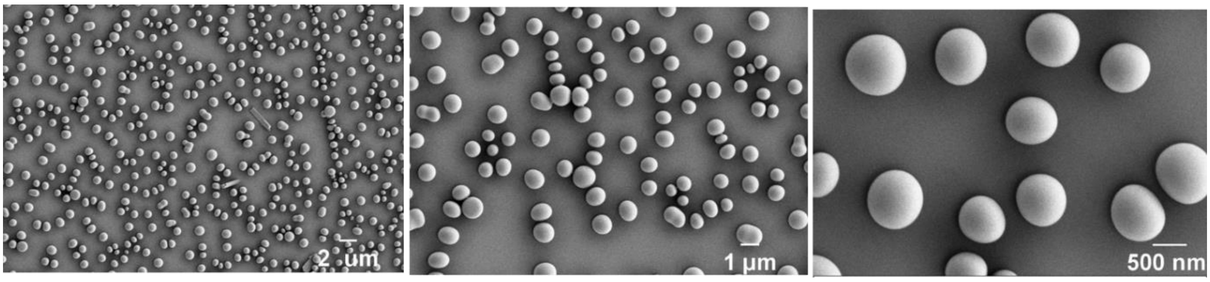

Figure S 23: SEM images of dropcasted sample of DPA-TCNB solution in ACN/EtOH (20/80%).

22. SEM images of DPA-TCNB suspension in ACN/EtOH/water (8:32:60%)

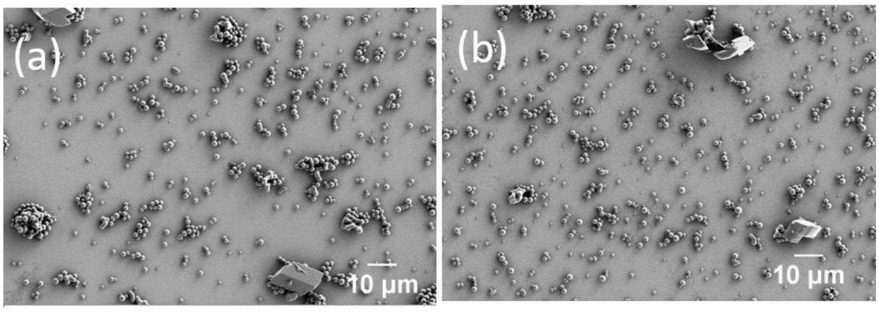

Figure S 24: SEM images of DPA-TCNB CT microspheres prepared in ACN/EtOH/water (8:32:60%) with crystalline particle in some regions.

23. Emission of DPA-TCNB suspension in ACN/EtOH/water (8:32:60%) recorded with time

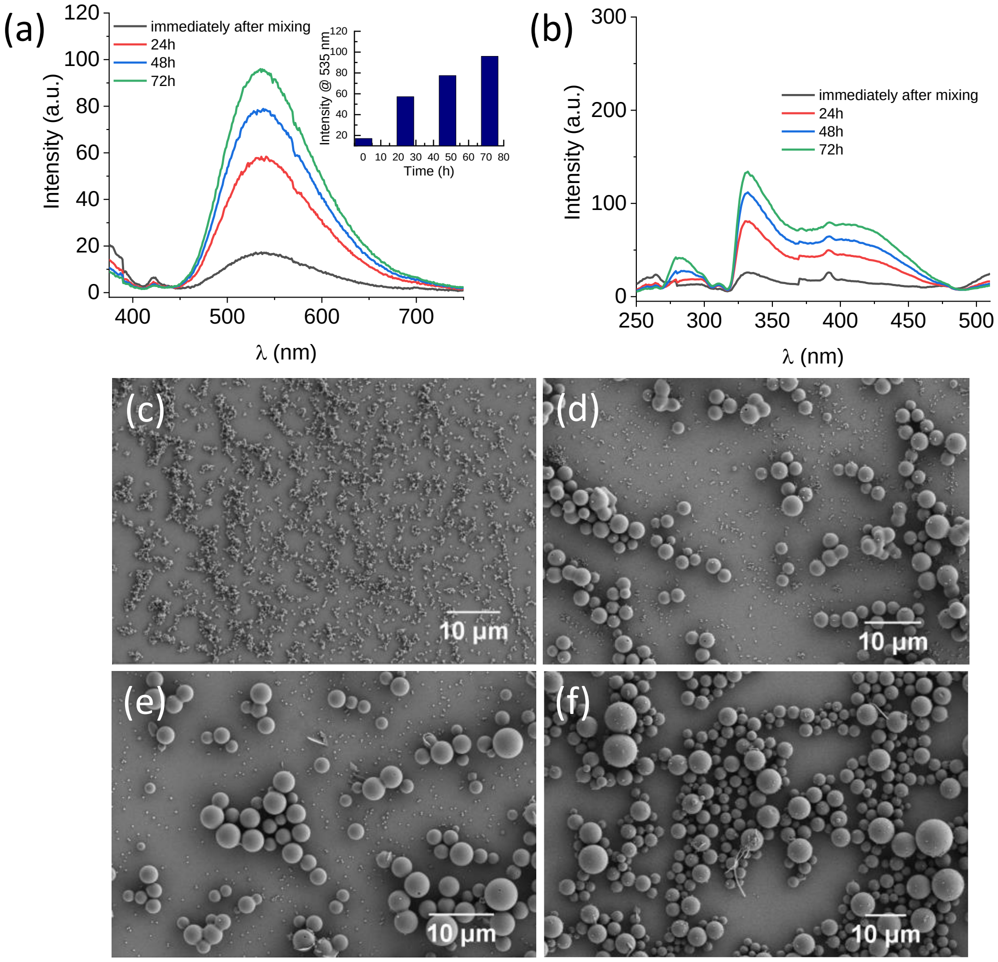

Figure S 25: Variation of (a) emission spectra for λ_{ex} 350 nm, inset: emission at λ_{em} 535 nm, and (b) excitation spectra for λ_{em} 535 nm of DPA-TCNB co-assembly in ACN/EtOH/water (8:32:60%), with time, respectively; SEM images recorded (c) immediately after preparation, (d) 24 h, (e) 48h, and (f) 72h after preparation.

24. SEM and Confocal microscopy images of TCNB

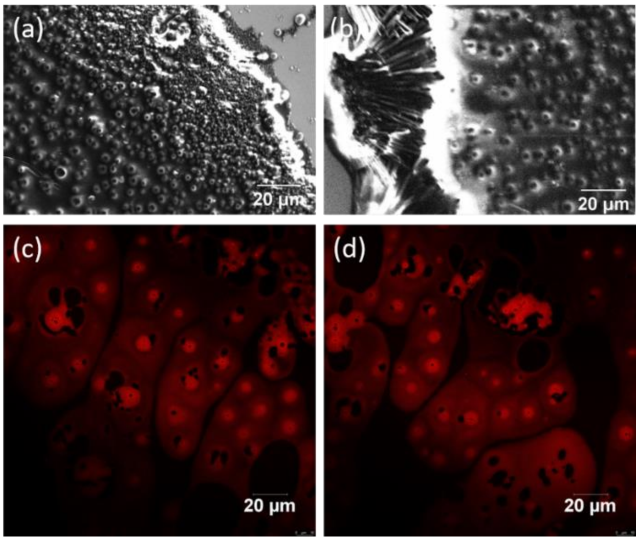

Figure S 26: (a, b) SEM images of TCNB prepared in ACN/EtOH/water (8:32:60%), (c,d) Confocal images of TCNB and Rhodamine mixture prepared in ACN/EtOH/water (8:32:60%).

25. Table S1 Crystal data and structure refinement for DPA-TCNB co-crystal.

Identification code	DPA-TCNB
Empirical formula	$C_{53}H_{37}N_{11}O_4$
Formula weight	891.93
Temperature/K	100.0
Crystal system	triclinic
Space group	P1
a/Å	10.1334(4)
b/Å	10.2002(3)
c/Å	11.4807(4)
α/°	93.3946(13)
β/°	99.9779(14)
γ/°	107.9484(13)
Volume/Å^3	1103.79(7)
Z	1
ρ_{calc}g/cm^3	1.342
μ/mm^{-1}	0.089
F(000)	464.0
Crystal size/mm^3	0.35 × 0.19 × 0.11
Radiation	Mo Kα (λ = 0.71073)
2Θ range for data collection/°	4.968 to 61.51
Index ranges	-14 ≤ h ≤ 14, -14 ≤ k ≤ 14, -16 ≤ l ≤ 16
Reflections collected	45138
Independent reflections	13673 [R_{int} = 0.0601, R_{sigma} = 0.0686]
Data/restraints/parameters	13673/4/621
Goodness-of-fit on F^2	1.011
Final R indexes [I>=2σ (I)]	R_1 = 0.0551, wR_2 = 0.1136
Final R indexes [all data]	R_1 = 0.0934, wR_2 = 0.1315
Largest diff. peak/hole / e Å^{-3}	0.38/-0.34
Flack parameter	-0.6(5)
CCDC	2155482

26. Single crystal and calculated Hirschfield surface of DPA-TCNB co-crystal

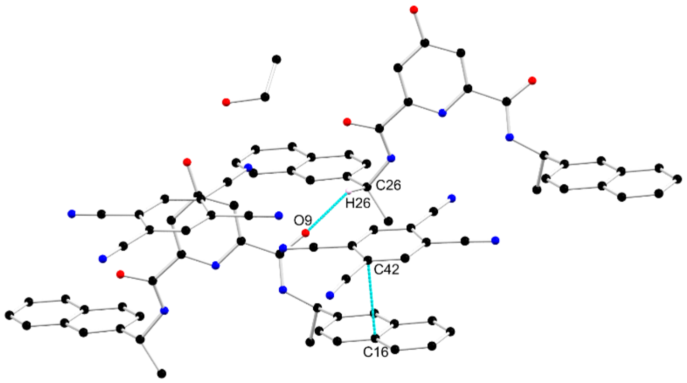

Figure S 27: Extended Structure of CT complex DPA-TCNB featuring π · · · π stacking and C-H···O interactions

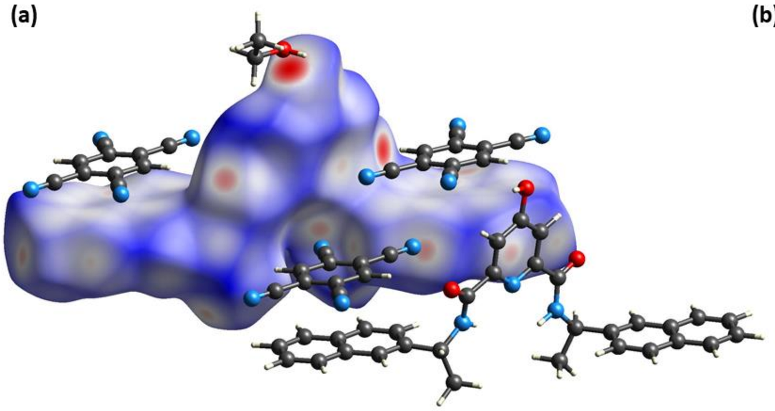

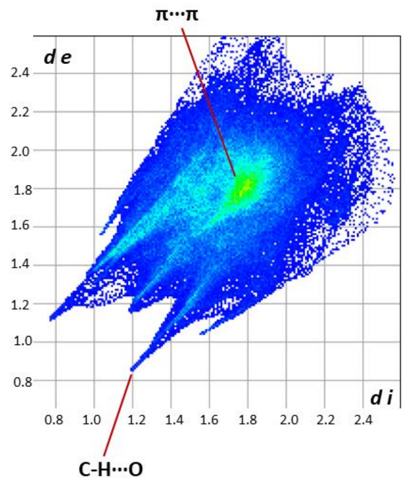

Figure S 28: (**a**) Calculated Hirshfield (d_{norm}) surface with normalised contact distance (D_{norm}) mapping for **DPA + TCNB** showing the main π···π and C-H···O interactions for each compound. Figures prepared with Crystal Explorer software, rendered with surface property in the range -0.65 to +1.35. [7-8] (**b**) Calculated Fingerprint plots from Hirshfield surface for **DPA + TCNB** showing the main π···π and C-H···O interactions for each compound.

27. Thermogravimetric analysis:

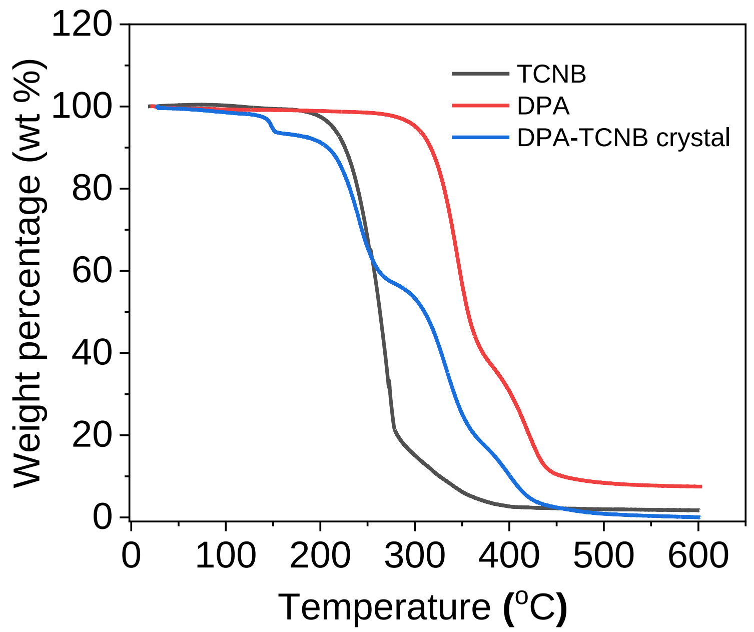

Figure S 29: TGA plot of DPA-TCNB crystal (blue line), only DPA (red line) and TCNB (black line).

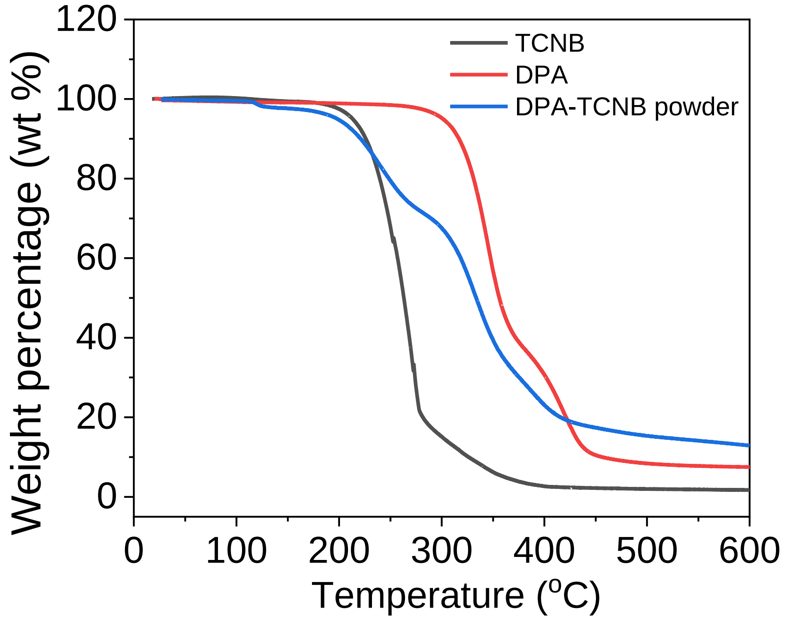

Figure S 30: TGA plot for DPA-TCNB powder (blue line), DPA powder (red line) and TCNB (black line).

28. NMR spectral comparison

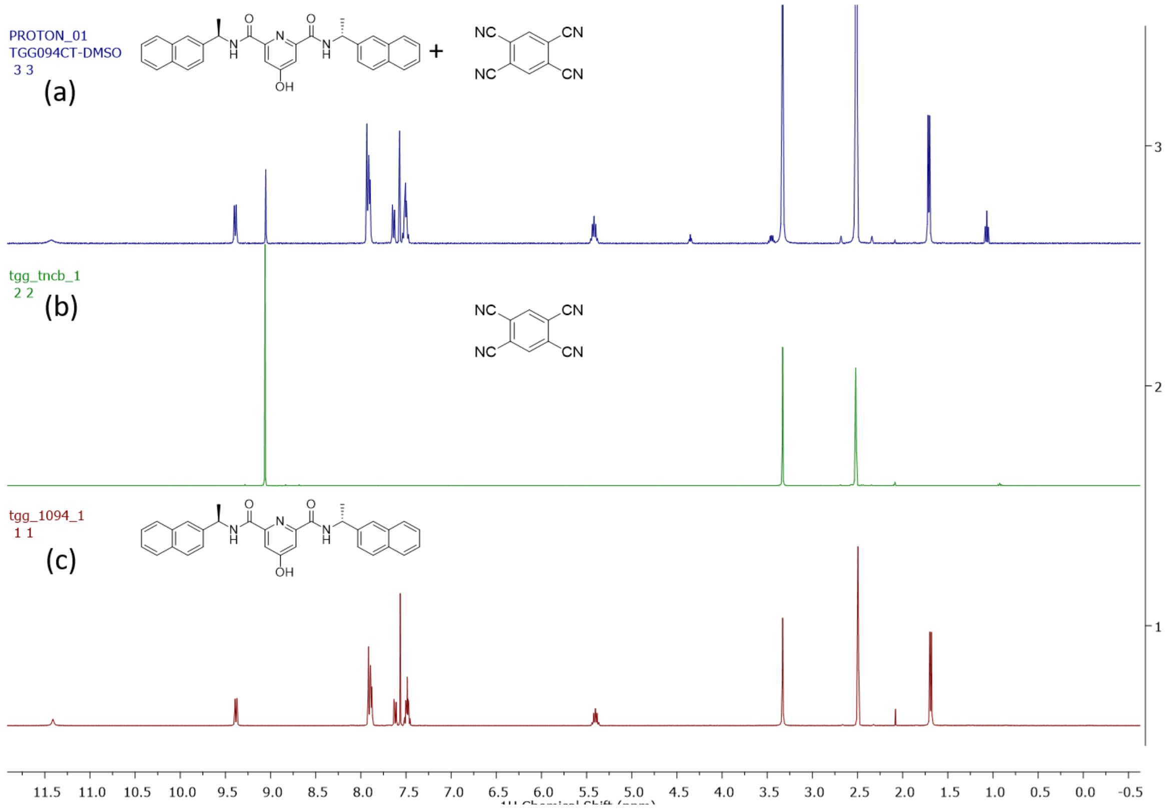

Figure S 31: NMR spectra in DMSO-D_6 for DPA+TCNB, TCNB, DPA, respectively from top to bottom.

29. Infrared spectral comparison

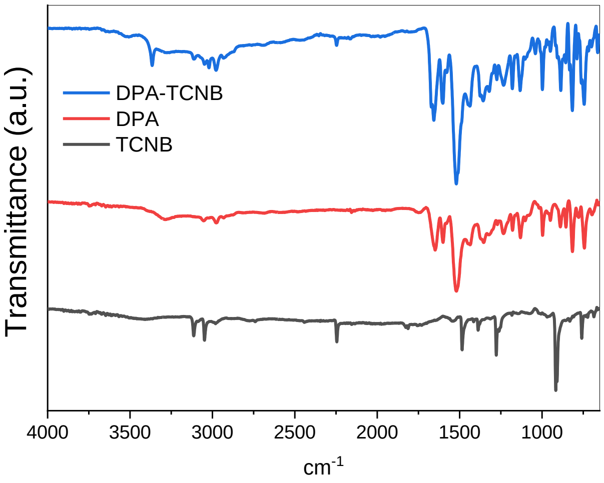

Figure S 32: Infrared spectra for DPA-TCNB powder (blue line), DPA (red line) and TCNB (black line).

30. Spectral overlap between emission spectra of DPA-TCNB CT co-assembly and Pyronin Y absorption

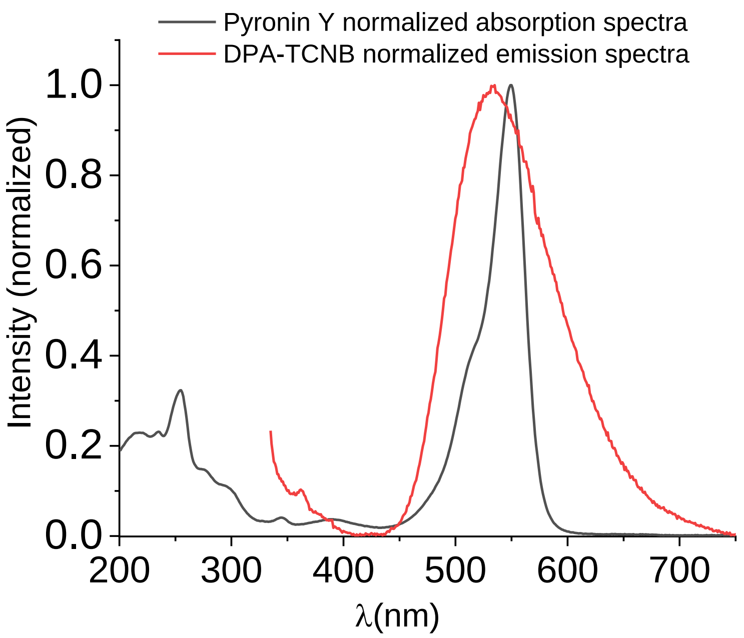

Figure S 33: Emission spectra of DPA-TCNB CT assembly and absorption spectra of Pyronin Y.

31. Comparison of emission and excitation spectra of Pyronin Y

Pyronin Y itself is very weakly emissive in aqueous medium on excitation at 280 nm or 325 nm, the intensity of Pyronin Y emission increased when encapsulated in DPA sphere, and it increased further in PYY-DPA-TCNB tri-component assembly due to energy transfer process.

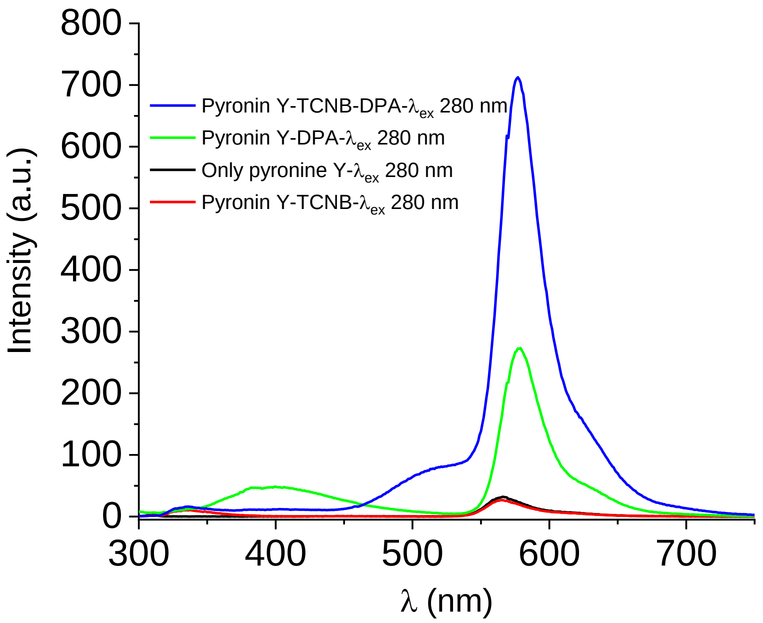

Figure S 34: Emission spectra of Pyronin Y (1.52 µM) alone, bi-component mixture of Pyronin Y (1.52 µM) and TCNB (1 mM) mixture, Pyronin y (1.52 µM) and DPA (1 mM) mixture, and tri-component mixture of Pyronin Y (1.52 µM), DPA (1 mM) and TCNB (1 mM) mixture in ACN/ EtOH/water (8:32:60%) mixture on λ_{ex} 280 nm.

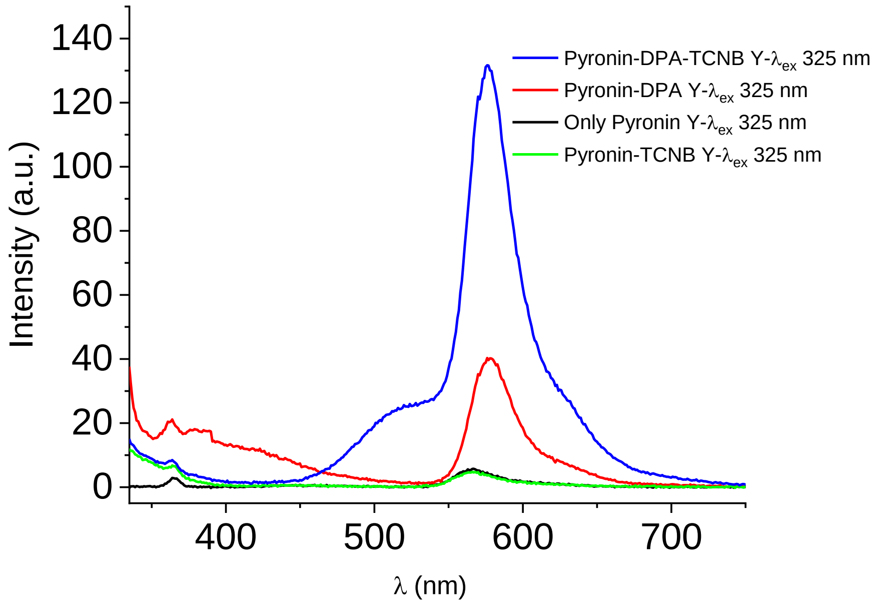

Figure S 35: Emission spectra of Pyronin Y (1.52 µM) alone, bi-component mixture of Pyronin Y (1.52 µM) and TCNB (1 mM) mixture, Pyronin y (1.52 µM) and DPA (1 mM) mixture, and tri-component mixture of Pyronin Y (1.52 µM), DPA (1 mM) and TCNB (1 mM) mixture in ACN/ EtOH/water (8:32:60%) mixture on λ_{ex} 325 nm.

32. Table S2: Lifetime data of CT emission at 535 nm

Sample	Lifetime(ns) λ_{ex} 340 nm, λ_{em} 535 nm	Average lifetime (ns)
DPA-TCNB (1 mM each)	22.76 (28.11%) + 84.36 (71.89%) χ^2 = 1.19	78.48
DPA(1 mM)-TCNB (1 mM)-PYY(0.62 µM)	12.2 (29.56%) + 73.75 (70.44%) χ^2 = 1.6	69.75
DPA(1 mM)-TCNB (1 mM)-PYY(1.55 µM)	9.94 (32.97%) + 64.62(67.03%) χ^2 = 1.94	60.77

33. Confocal images of DPA-TCNB and DPA-TCNB-PYY

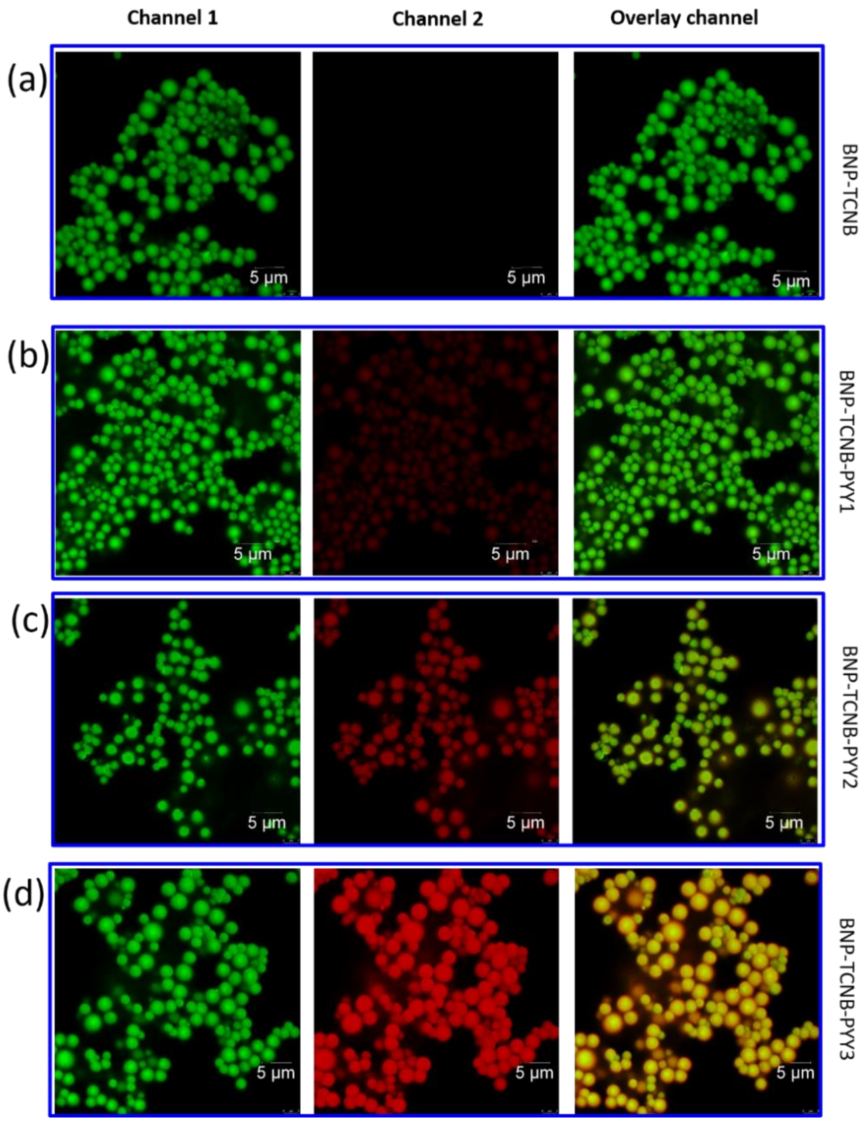

Figure S 36: Confocal microscopic images of DPA-TCNB (a) and DPA-TCNB-PYY co-assembly with PYY concentration 0.22 µM, 0.88 µM, 1.78 µM, respectively (b-d).

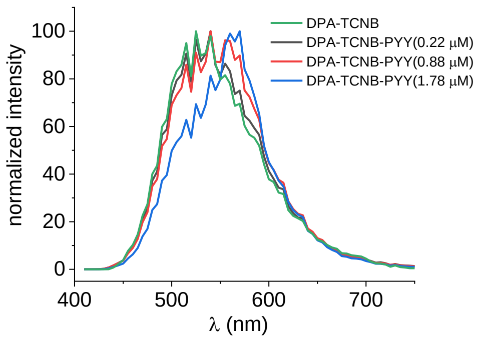

Figure S 37: Emission spectra recorded from confocal images for DPA-TCNB and DPA-TCNB-PYY with PYY concentration 0.22 µM, 0.88 µM, 1.78 µM.

34. Confocal image of DPA+TCNB microsphere in 3D mode

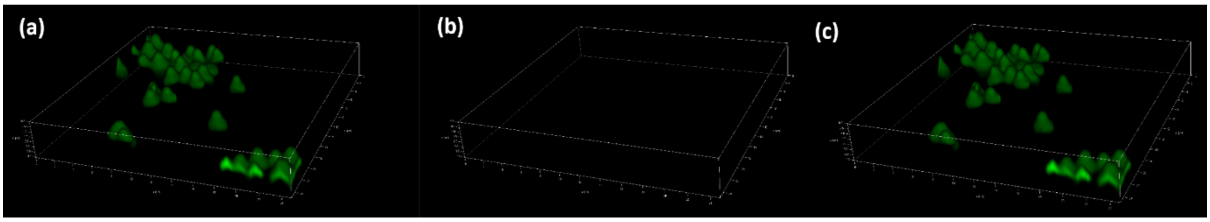

Figure S 38: 3D image of DPA+TCNB (1 mM each), (a) green channel, (b) red channel, and (c) overlay of both channels.

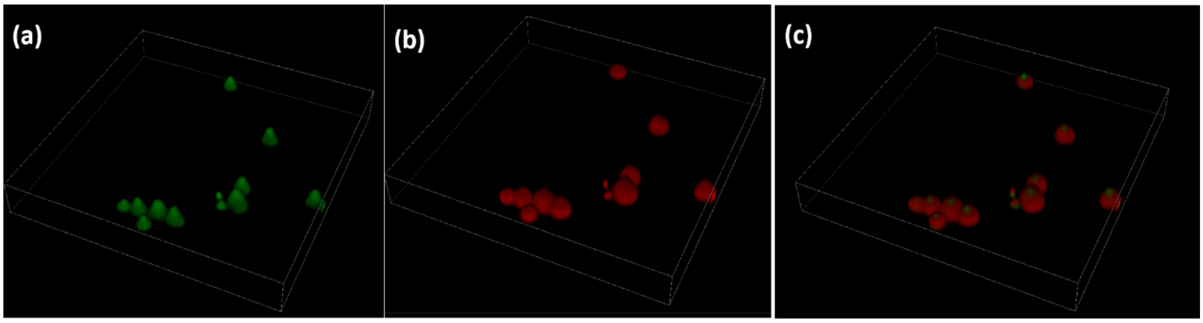

Figure S 39: 3D image of DPA+TCNB +PYY (1mM /1mM/1.78 µM), (a) green channel, (b) red channel, and (c) overlay of both channels.

35. References

1. Bruker APEX-3, Bruker-AXS Inc., Madison, WI, 2016.
2. SADABS, Bruker-AXS Inc., Madison, WI, 2016.
3. G. M. Sheldrick, SHELXT - Integrated space-group and crystal-structure determination, *Acta Crystallogr. Sect.* A. 2015, **71**, 3-8.
4. G. M. Sheldrick, Crystal Structure Refinement with SHELXL, *Acta Crystallogr. Sect. C*. 2015, **71**, 3-8.
5. Gaussian 16, Revision C.01, M. J. Frisch, et. al.
6. S. J. Bradberry, A. J. Savyasachi, R. D. Peacock and T. Gunnlaugsson, *Faraday Discuss*, 2015, **185**, 413-431.
7. *CrystalExplorer Version 3.1,* S.K. Wolff, D.J. Grimwood, J.J. McKinnon, M.J. Turner, D. Jayatilaka, M.A. Spackman, University of Western Australia, 2012.
8. M.A. Spackman and D. Jayatilaka, Hirshfeld Surface Analysis, *CrystEngComm,* 2009, **11** , 19-32.
